# Resilience of a stochastic generalized Lotka–Volterra model for microbiome studies

**DOI:** 10.3934/mbe.2025056

**Published:** 2025-05-14

**Authors:** Tuan A. Phan, Benjamin J. Ridenhour, Christopher H. Remien

**Affiliations:** 1 Institute for Modeling Collaboration and Innovation, University of Idaho, Moscow, ID 83844, USA; 2 Department of Mathematics and Statistical Science, University of Idaho, Moscow, ID 83844, USA

**Keywords:** stochastic gLV, resilience measures, instantaneous return rate, average return rate, convergence rate, asymptotic resilience

## Abstract

Microbial communities are constantly challenged by environmental stochasticity, rendering time-series data obtained from these communities inherently noisy. Traditional mathematical models, such as the first-order multivariate autoregressive (MAR) model and the deterministic generalized Lotka–Volterra model, are no longer suitable for predicting the stability of a microbiome from its time-series data, as they fail to capture volatility in the environment. To accurately measure microbiome stability, it is imperative to incorporate stochasticity into the existing mathematical models in microbiome research. In this paper, we introduce a stochastic generalized Lotka–Volterra (SgLV) system that characterizes the temporal dynamics of a microbial community. To study this system, we developed a comprehensive theoretical framework for calculating four resilience measures based on the SgLV model. These resilience metrics effectively capture the short- and long-term behaviors of the resilience of the microbiome. To illustrate the practical application of our approach, we demonstrate the procedure for calculating the four resilience measures using simulated microbial abundance datasets. The procedural simplicity enhances its utility as a valuable tool for application in various microbial and ecological communities.

## 1. Introduction

Microbiome dynamics are inherently stochastic. They are not just the assemblage of living microorganisms present in a defined environment, but also encompass the entire spectrum of molecules, structural elements, metabolites, and mobile genetic elements, produced by these microorganisms, known as their theater of activity [1]. Consequently, interpreting the causal link between microbiomes and human health, a critical aspect of microbiome research [2, 3], remains challenging.

Environmental fluctuations can induce variability in interactions within a microbiome and between microbes and their environment or host. For instance, some microbial species may exhibit varying levels of sensitivity to temperature, impacting their interactions with other microbes or with the environment/host depending on the ambient temperatures. This variability introduces errors in the estimation of microbial interactions, which, in turn, affect our ability to understand the drivers of dynamics. It becomes imperative to translate microbial interactions, which govern the microbiome’s population dynamics, into a high-level property of a microbiome, such as its resilience.

**Figure 1.**
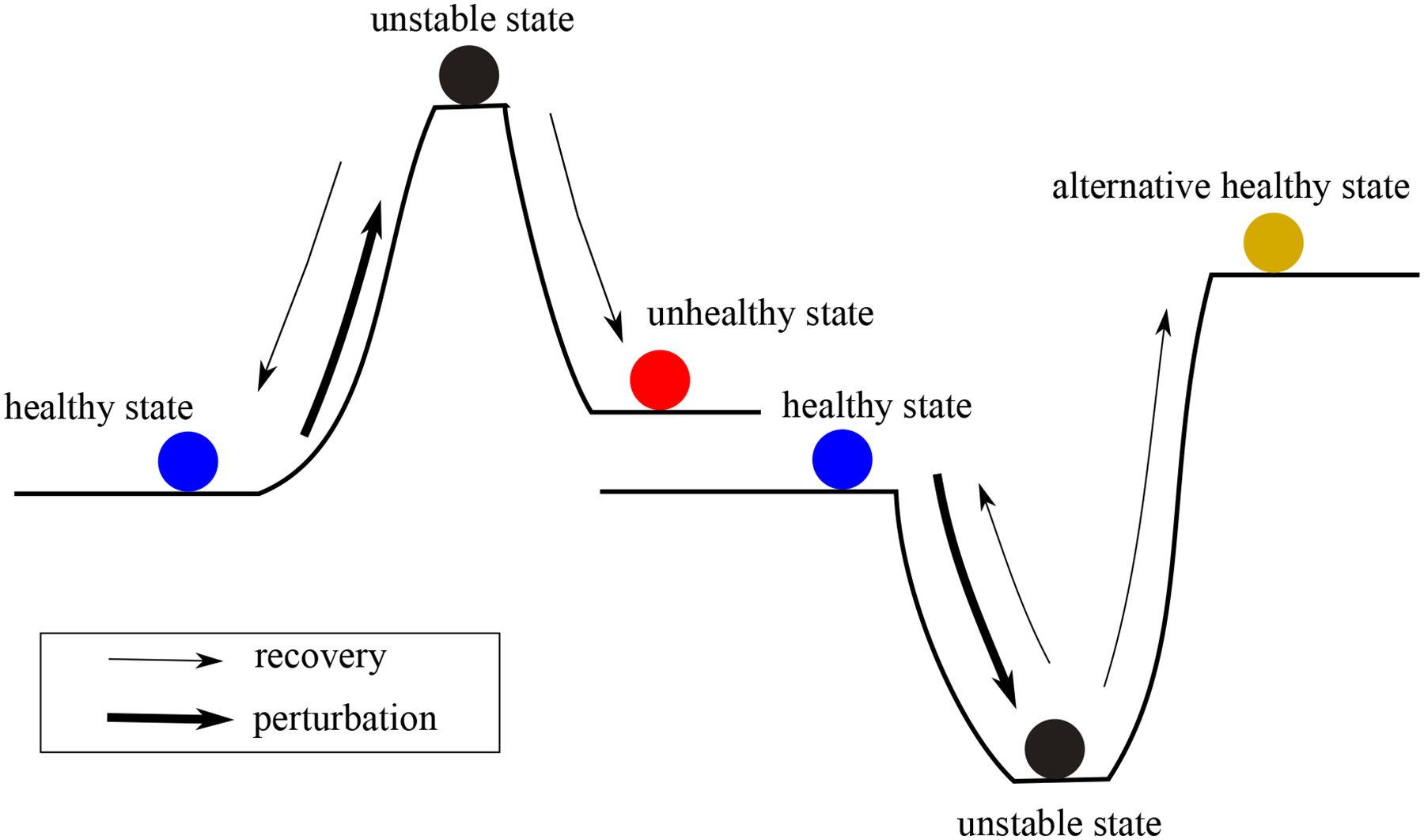
Conceptual illustration of the resilience of a microbiome.

Theories from dynamical systems, adapted from macroecology, offer insight into understanding the dynamics of the microbiome in different environments [4]. The resilience of a microbiome, i.e., its ability to maintain and recover function in the face of perturbations, such as those caused by antibiotics or opportunistic pathogens, can be studied by employing the concept of stability in dynamic systems. Microbiomes that more readily return to stationarity (i.e., when the dynamics are captured by white noise about an equilibrium) when faced with perturbations are considered to be more resilient. Figure 1 illustrates the concept of the resilience of the microbiome. The impact of a perturbation, classified as a “pulse” or a “press,” on a microbial community is determined by the duration and magnitude of the perturbation [5]. Following a perturbation, a microbial community in a healthy state (eubiosis) may either transition to an unstable and transient state or recover to its initial state. In some instances, the perturbation might drive the community towards the formation of an alternative healthy state or an unhealthy stable state (dysbiosis) associated with disease. Recent empirical evidence supports these theoretical concepts. Studies of gut microbiota dynamics have revealed intrinsic stability patterns and catastrophic shifts following antibiotic perturbations [6, 7]. Moreover, mechanistic research has elucidated how commensals regulate host immunity, such as the suppression of retinoic acid synthesis to control IL-2 activity and prevent dysbiosis [8]. Beyond the gut, investigations across different body sites demonstrate community-specific resilience patterns, from vaginal microbiome’s stability [9] to oral ecosystems [10]. Building on these findings, recent comprehensive reviews have established resilience as the microbiota’s universal capacity to resist and recover from perturbations [11, 12], with longitudinal studies revealing personalized resilience features, such as the individual-specific stability observed during the Mars500 mission [13]. Therefore, understanding how a microbiome responds to perturbations is crucial to preventing disease states and promoting health.

The concept of resilience has been intensively studied in macroecological systems. In fact, the ecological term “resilience” was originally coined by Holling 1973 [14]. In this seminal article, the author defined resilience as a “measure of the persistence of ecosystems and their ability to absorb change and disturbance and still maintain the same relationships between populations or state variables.” This concept was subjected to a comprehensive examination by Pimm and Lawton 1977 [15], DeAngelis 1980 [16], and Cottingham and Carpenter 1994 [17]. The index most frequently used to calculate resilience in ecosystems is based on the eigenvalues of an ordinary differential equation system near its equilibrium. Resilience is computed as the negative reciprocal of the maximum eigenvalue, providing a measure of the time it takes the ecosystem to recover from a perturbation. Subsequently, Neubert and Caswell 1997 [18] introduced four quantities (asymptotic resilience, reactivity, and two quantities of the amplification envelope) to measure the long- and short-term responses of an ecosystem to pulse perturbations. More recently, Arnoldi et al., [19, 20] attempted to unify all previous resilience measures for a community, making them more practical by extending the analysis of ecosystem responses to perturbations on all time scales.

All the previously mentioned definitions of resilience are based on the theory of ordinary differential equations, in which an equilibrium state is assumed to be a fixed point of a deterministic dynamic system. However, due to continuously fluctuating environments, equilibrium states may not be fixed points but fluctuating stationary distributions. To address this issue, Ives 1995 [21] proposed a definition of stochastic resilience based on a discrete-time linear stochastic model. This article used the process error, modeled as a sequence of normal random variables with a mean of 0 and a constant variance, to account for the variability in interactions between species. Subsequently, Ives et al., 2003 [22] extended this definition to three additional stability measures using a first-order discrete-time multivariate autoregressive model (MAR). Buckwar and Kelly 2014 [23] redefined the notions of asymptotic resilience, reactivity, and the amplification envelope, proposed by Neubert and Caswell 1997 [18], in terms of the second moment of a linear Ito stochastic differential equation system, with the goal of investigating the effect of persistent stochastic perturbations on a deterministic system.

Over the past decade, the conceptualization of resilience within microbiome research has evolved significantly through the integration of stochastic modeling approaches. Zaoli and Grilli 2021 [26] analyzed longitudinal human gut microbiome data, revealing that while most operational taxonomic units (OTUs) exhibit stable dynamics with fluctuations around a constant average, a minority display nonstationary behavior with abrupt transitions in abundance, potentially indicating shifts between alternative stable states. This study underscored the importance of considering stochastic fluctuations and the potential for multiple stable states in microbial communities.

Building on this foundation, Wolff et al., 2023 [27] investigated the daily dynamics of intraspecific genetic variation in the human gut microbiome. They discovered that approximately 80% of the strains analyzed exhibited abundance fluctuations that could be predicted using a stochastic logistic model, suggesting that the stability and macroecological properties of the human gut microbiome emerge at the strain level. Hayashi et al., 2024 [24] conducted time-series analyses of experimental microbiomes, demonstrating that both deterministic and stochastic ecological processes drive the divergence of alternative states. This work highlighted the role of stochasticity in generating multiple stable states within microbial communities. Concurrently, Ponciano et al., 2024 [25] emphasized the necessity of integrating stochastic models to predict a population’s persistence probabilities within the vaginal microbiome, advocating for a shift from static snapshots to dynamic modeling approaches. Collectively, these studies illustrate a progressive shift towards embracing stochastic modeling to more accurately capture the resilience and dynamic behavior of microbiome communities.

Although well studied, existing resilience measures are inadequate for microbiome studies because stochasticity, known to affect the stability of a dynamical system, does not influence these measures. Therefore, there is a pressing need to incorporate stochasticity, such as environmental noise and uncertainty, into the mechanistic models used to identify causal mechanisms using time-series microbiome data. In nature, the environment often fluctuates randomly and without memory, making white noise an excellent candidate to account for environmentally driven stochastic effects. Given the apparent need for such a model, we propose a stochastic generalized Lotka–Volterra (SgLV) model to describe the temporal dynamics of a community of microbial species. Stochasticity is induced in the model by adding white noise to the model parameters. Then, based on the linearization of our proposed SgLV model, we develop resilience measures to quantify resilience that can be applied to time-series microbiome data.

In our modeling framework, we represent environmental stochasticity using white noise. Specifically, we modify a constant parameter, such as α, to include stochastic fluctuations by redefining it as $\overset{‾}{\alpha }=\alpha +\tau \frac{dW}{dt}$, where W(t) is standard Brownian motion, and τ represents the intensity of noise. Consequently, $\overset{‾}{\alpha }$ transforms from a fixed parameter into a stochastic process. Within a sufficiently small time interval [0,t], we approximate the term $\frac{dW}{dt}$ by $\frac{W(t)}{t}$, which follows a normal distribution $N\left(0,\frac{1}{t}\right)$. Thus, each time we simulate the model, the value of $\overset{‾}{\alpha }$ will be chosen from the normal distribution $N\left(\alpha ,\alpha +\tau^{2}/t\right)$. This way of incorporating noise is more realistic than that in Ives et al., 2003 [22], since the variance of the noise is not constant but changing over time.

The foundational work by Ives et al., 2003 [22] established critical methodologies for analyzing ecological community stability using discrete-time multivariate autoregressive (MAR) models, providing robust methods for estimating stability and ecological interactions from empirical time-series data. Their pioneering approach revealed important relationships between species interactions and environmental variability, significantly advancing the quantitative study of ecological dynamics. In our manuscript, we extend this foundational framework from discrete time to continuous time by developing stochastic generalized Lotka–Volterra models, which are particularly suited to capturing microbiome dynamics characterized by continuous growth and interaction processes. Moreover, we propose novel resilience metrics specifically tailored to microbiome community dynamics, including measures capable of quantifying recovery following perturbations and assessing how stochasticity influences the pace of community restoration. Thus, while conceptually rooted in the seminal work of Ives et al., 2003 [22], our model provides significant advancements optimized for addressing the unique complexities inherent in microbiome research.

The remainder of the paper is organized into three sections. First, we develop a general theoretical framework on how to calculate four resilience measures for the proposed SgLV model. Second, we demonstrate the practicality of our framework by applying it to a stochastic two-species competitive model to illustrate the effects of environmental noise on the resilience of the stochastic model. Lastly, we have a brief discussion on the possibilities of applying our framework to real microbiome data.

## 2. General theoretical framework

**Figure 2.**
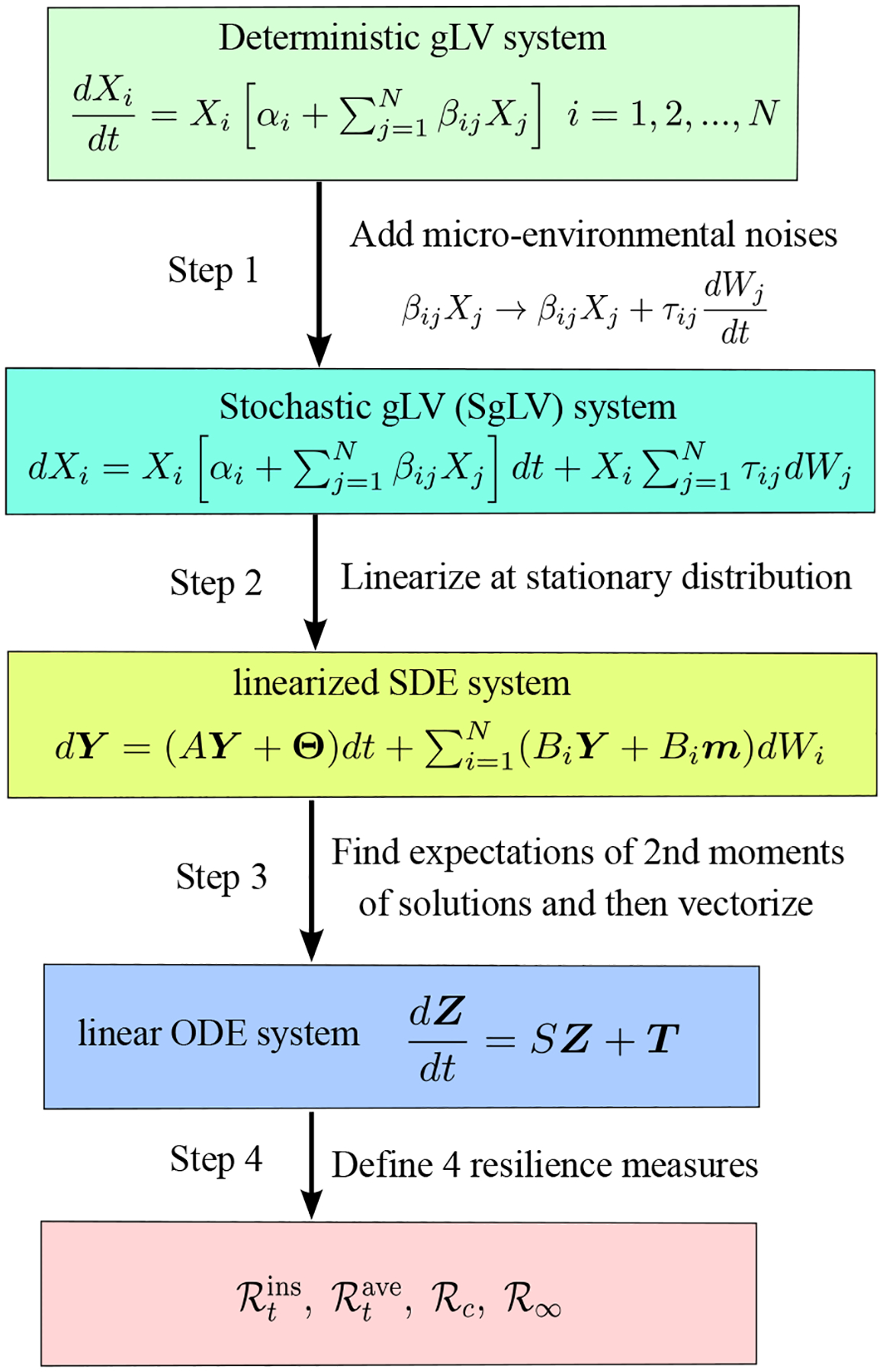
Four-step scheme of the method of calculating the resilience of a SgLV.

In this section, we develop a general theoretical framework to calculate four resilience measures that quantify the recovery of a microbial community from pulse perturbations (single shocks or perturbations of relatively short duration). In contrast to previous works that used ordinary differential equation (ODE) systems [18–20] and/or stochastic discrete-time systems [21, 22], we use a stochastic differential equation (SDE) system to describe the temporal dynamics of species within a microbiome community and then study its transient dynamics under different pulse perturbations. We utilize white noise to represent random environmental fluctuations without memory to capture the fluctuations observed in microbiomic time-series data. We consider them to be the effects of persistent environmental perturbations. Mathematically, we add white noise, described as the “derivative of Brownian Motion,” to a deterministic gLV model. We then obtain a non-linear stochastic gLV model. Next, we linearize this nonlinear SDE system at its positive stationary distribution to get a linear SDE system. On the basis of two works by Arnoldi et al., [19, 20], we indirectly establish four resilience measures, which are the *instantaneous return rate* $\left({ℛ}_{t}^{\text{ins}}\right)$, *average return rate* $\left({ℛ}_{t}^{\text{ave}}\right)$, *asymptotic resilience* $\left({ℛ}_{\infty }\right)$, *and the convergence rate* $\left({ℛ}_{c}\right)$, of our stochastic gLV model, through the second moment of solutions of the corresponding linearized SDE system. These four resilience quantities, in which the first two are functions of time and the last two are just numbers, measure how strong the recovery dynamics of our original stochastic system is after a pulse perturbation is applied. Figure 2 shows the scheme of the theoretical method used to calculate the resilience of a stochastic generalized Lotka–Volterra system of a microbial community of N species. The details of this method will be presented in the following four subsections.

### 2.1. Model setup and notation

The widely used mechanistic model for microbiome studies is the deterministic generalized Lotka–Volterra model, which is given by

(2.1) $$\frac{dX_{i}}{dt}=X_{i}\left[\alpha_{i}+\sum_{j=1}^{N}\beta_{ij}X_{j}\right],\;i=1,\cdots ,N,$$

in which $X_{i}$ is the abundance of species i in a microbiome community. The parameter $\alpha_{i}$ represents the intrinsic growth rate of species i. The interaction between species i and species j(j≠i) is captured by the parameter $\beta_{ij}$. When i=j, the parameter $\beta_{ij}$ takes the form $\beta_{ii}=-\alpha_{i}c_{i}<0$ where $c_{i}$ is the coefficient of negative intraspecific interaction representing the inverse of the carrying capacity of the species i in isolation.

To characterize the microenvironmental fluctuations and noises in pairwise interactions between species within a microbiome community, we propose a general *stochastic generalized Lotka–Volterra* (SgLV) model that takes the following form

(2.2) $$dX_{i}=X_{i}\left[\alpha_{i}+\sum_{j=1}^{N}\beta_{ij}X_{j}\right]dt+X_{i}\sum_{j=1}^{N}\tau_{ij}dW_{j},\;i=1,\cdots ,N,$$

Notice that we perturbed each interaction coefficient $\beta_{ij}$ in the system (2.1) by replacing each term $\beta_{ij}X_{j}$ with the term $\beta_{ij}X_{j}+\tau_{ij}\frac{dW_{j}}{dt}(i,j=1,\cdots ,N)$ where ${\left(W_{1},\cdots ,W_{N}\right)}^{T}$ is a standard Brownian motion of N dimensions.

For convenience, we introduce our notation that will be used throughout this paper. First, we need to specify an appropriate completed filtered probability space. Let $\Omega =\left\{\omega \in C\left(R,R^{N}\right),\omega (0)=0\right\}$, let ℱ be the Borel σ-algebra on Ω, and let P be the measure induced by $\{W=W(t){\}}_{t\in R}$, a two-sided N-dimensional Brownian motion. Then the elements of Ω will be identified with paths of the N-dimensional Brownian process ω(t)=W(t,ω). Now we consider the P-completion of ℱ, also denoted by ℱ, that is, ℱ contains all P-null sets. The filtration ${ℱ}_{t}$ is given by the canonical filtration generated by the Brownian motion $\{W(t){\}}_{t\geq 0}$ completed by all P-null sets of ℱ. Denote the probability measure given by the extension of P to the completed ℱ again by P. Therefore, a completed filtered probability space $\left(\Omega ,ℱ,{\left\{{ℱ}_{t}\right\}}_{t\geq 0},P\right)$ is obtained. Next, consider the SgLV (2.2) whose solutions take values in $[0,\infty {)}^{n}$ and describe the abundance dynamics of N interacting species $X(t)≔{\left(X_{1}(t),\cdots ,X_{N}(t)\right)}^{T},t\geq 0$, in a microbial community. We write

$$R^{N}≔\left\{{\left(x_{1},\cdots ,x_{N}\right)}^{T}:x_{1}\in R,\cdots ,x_{N}\in R\right\},$$

$$R_{+}^{N}≔\left\{{\left(x_{1},\cdots ,x_{N}\right)}^{T}\in R^{N}:x_{1}\geq 0,\cdots ,x_{N}\geq 0\right\},$$

$$R_{+}^{N,\circ }≔\left\{{\left(x_{1},\cdots ,x_{N}\right)}^{T}\in R^{N}:x_{1}>0,\cdots ,x_{N}>0\right\},$$

$$f_{i}=f_{i}(X(t))≔\alpha_{i}+\sum_{j=1}^{N}\beta_{ij}X_{j}\;(\text{the per-capita growth rate of the species}\;i),$$

$$\Gamma ≔{\left(\tau_{ij}\right)}_{N\times N},\;\text{and}\;\Sigma ≔\Gamma \Gamma^{T}={\left(\sigma_{ij}\right)}_{N\times N}\;\text{where}\;\sigma_{ij}=\sum_{k=1}^{N}\tau_{ik}\tau_{jk}.$$

From now on, the stochastic process given by the solution of the system (2.2) will be denoted by X or $(X(t){)}_{t\geq 0}$. We use $P_{x}$ to denote the probability law on Ω when the solution path starts at $x={\left(x_{1},\cdots ,x_{n}\right)}^{T}$ and $E_{x}$ is the expectation corresponding to $P_{x}$.

We use the norm $\|x\|≔\sum_{i=1}^{N}\left|x_{i}\right|$ in $R^{N}$ where $x={\left(x_{1},\cdots ,x_{N}\right)}^{T}$. For ${\left(u_{1},\cdots ,u_{N}\right)}^{T}\in R^{N}$, we let $\overset{N}{\underset{i=1}{⋀}}u_{i}≔\text{min}\left\{u_{1},\cdots ,u_{N}\right\}$ and $\overset{N}{\underset{i=1}{⋁}}u_{i}≔\text{max}\left\{u_{1},\cdots ,u_{N}\right\}$.

### 2.2. Persistence and extinction

Based on the excellent work of Hening and Nguyen 2018 [32–34], we briefly present, without proofs, sharp sufficient conditions for both persistence and extinction for SgLV (2.2) and its corresponding deterministic counterpart.

First, we consider the corresponding deterministic gLV system of the stochastic system (2.2) given by

(2.3) $$\frac{dX}{dt}=h\left(X\right),$$

where $h(X)={\left(h_{1}(X),\cdots ,h_{N}(X)\right)}^{T}$, and $h_{i}(X)≔X_{i}\left[\alpha_{i}+\sum_{j=1}^{N}\beta_{ij}X_{j}\right],i=1,\cdots ,N$. We define persistence and extinction for the deterministic system (2.3) as follows.

**Definition 2.1.** *The system* (2.3) *is persistent if, for any initial value* X(0) *in* $R_{+}^{N,\circ }$, *each component of the solution* X(t) *satisfies* $\underset{t\to \infty }{\text{lim}\;\text{inf}}\;X_{i}(t)>0$ *for all* i=1,⋯,N.

**Definition 2.2.** *For any initial value* $X(0)\in R_{+}^{N,\circ }$, *the species* $X_{i}$ *is said to go extinct if* $\underset{t\to \infty }{\text{lim}\;\text{sup}}\;X_{i}(t)=0$.

In a deterministic setting, in order to investigate the persistence and extinction of the system (2.3) (i.e., we wish to know which species persist or which species go extinct in this system), we look at the set 𝒩, which is the set of all the equilibria of the deterministic system (2.3) on the boundary $\partial R_{+}^{N}≔R_{+}^{N}\setminus R_{+}^{N,\circ }$, as well as the unique positive equilibrium $E^{*}$ in $R_{+}^{N,\circ }$. Clearly, 𝒩≠∅ since $0=(0,\cdots ,0{)}^{T}\in 𝒩$. Then, for each $E\in 𝒩\cup \left\{E^{*}\right\}$, we compute the eigenvalues $\lambda_{1}(E),\cdots ,\lambda_{N}(E)$ of the variational matrix ${\left.\frac{dh}{dX}\right|}_{X=E}$. Hence, we have the following theorem about the persistence of the system (2.3).

**Theorem 2.1.** *The microbial community described by the system* (2.3) *becomes persistent, i.e., the abundance of each species is positive, if all the equilibria on the boundary* $\partial R_{+}^{N,\circ }$ *are unstable, which means that*

(2.4) $$\underset{i=1,\cdots ,N}{\text{max}}Re\;\lambda_{i}\left(E\right)>0\;\text{for each}\;E\in 𝒩,$$

This is equivalent to the fact that the unique positive equilibrium $E^{*}$ is locally stable (i.e., all the eigenvalues of the linearized system at the positive equilibrium have non-positive real parts). If the deterministic system (2.3) is not persistent, we would like to track which species go extinct and which species survive. Indeed, for $0\neq E={\left(x_{1,E},\cdots ,x_{N,E}\right)}^{T}\in 𝒩,1\leq n_{1}<\cdots <n_{k}\leq N$ exist such that $x_{n_{i},E}>0$ for all i=1,⋯,k and $x_{j,E}=0$ for all $j\in \{1,\cdots ,N\}\setminus \left\{n_{1},\cdots ,n_{k}\right\}$. Let

$$R_{+}^{E}≔\left\{\left(x_{1},\cdots ,x_{N}\right)\in R_{+}^{N}:x_{i}=0\;\text{for}\;i\in I_{E}^{c}\right\},$$

where $I_{E}≔\left\{n_{1},\cdots ,n_{k}\right\}$ and $I_{E}^{c}=\{1,\cdots ,N\}\setminus \left\{n_{1},\cdots ,n_{k}\right\}$. If E=0, then $R_{+}^{0}=\{0\}$. Let

$$R_{+}^{E,\circ }≔\left\{\left(x_{1},\cdots ,x_{N}\right)\in R_{+}^{N}:x_{i}=0\;\text{for}\;i\in I_{E}^{c}\;\text{and}\;x_{i}>0\;\text{for}\;i\in I_{E}\right\}$$

and $\partial R_{+}^{E}=R_{+}^{E}\setminus R_{+}^{E,\circ },$. Now we make the following assumption.

**Assumption 1.** *If a* E∈𝒩 *exists such that*

$$\underset{i\in I_{E}^{c}}{\text{max}}\;\text{Re}\;\lambda_{i}(E)<0,$$

*If* $R_{+}^{E}\neq \{0\}$, *then suppose further that for any* $E^{\prime }\in {𝒩}_{E}$, *we have*

$$\underset{i\in I_{E}}{\text{max}}\;Re\;\lambda_{i}\left(E^{\prime }\right)>0,$$

*where* ${𝒩}_{E}≔\left\{E^{\prime }\in 𝒩:R_{+}^{E^{\prime }}\subseteq \partial R_{+}^{E}\right\}$.

Define

$${𝒩}^{1}≔\left\{E\in 𝒩:E\;\text{satisfies Assumption}\;1\right\}\;\text{and}\;{𝒩}^{2}≔𝒩\setminus {𝒩}^{1}.$$

To determine exactly which species go extinct, we need an additional assumption ensuring that equilibria that are not in ${𝒩}^{1}$ are unstable.

**Assumption 2.** *Either* ${𝒩}^{2}=\emptyset$ *or, for any* $E^{\prime }\in {𝒩}^{2},\underset{i=1,\cdots ,N}{\text{max}}Re\lambda_{i}\left(E^{\prime }\right)>0$.

**Theorem 2.2.** *If Assumptions 1 and 2 are satisfied and ${𝒩}^{1}\neq \emptyset$*, *then for any* $E\in {𝒩}^{1}$, *the species* $X_{i}(t)$ *converges to* 0 *with the convergence rate* $\lambda_{i}(E)$ *for any* $i\in I_{E}^{c}$.

Next, we consider the stochastic system (2.2). We make a standing assumption that will be used throughout this section.

**Assumption 3.** The noise intensity matrix Γ *of the system* (2.2) *satisfies the condition that the matrix* $\Sigma ≔\Gamma \Gamma^{T}={\left(\sigma_{ij}\right)}_{N\times N}$ *is a positive-definite matrix. Furthermore*, $c={\left(c_{1},\cdots ,c_{N}\right)}^{T}\in R_{+}^{N,\circ }$ *and* δ>0 *exist such that for sufficiently large* ‖X‖, *we have*

(2.5) $$\frac{\sum_{i=1}^{N}c_{i}X_{i}f_{i}(X)}{1+c^{T}X}-\frac{\sum_{i,j=1}^{N}\sigma_{ij}c_{i}c_{j}X_{i}X_{j}}{2{\left(1+c^{T}X\right)}^{2}}+\delta \left(1+N+\sum_{i=1}^{N}\left|f_{i}\left(X\right)\right|\right)<0.$$

Since the drift functions $X_{i}f_{i}(X(t))$ are locally Lipschitz functions on $R^{N}$ for any i=1,⋯,N and the diffusion functions in the system (2.2) are linear, for each initial value in $R^{N}$, the system (2.2) has a unique almost surely (a.s.) continuous solution X=X(t) up to the explosion time

$$\tau_{e}=\text{inf}\left\{t>0:\overset{N}{\underset{i=1}{⋀}}X_{i}(t)=-\infty \;\text{or}\;\overset{N}{\underset{i=1}{⋁}}X_{i}(t)=\infty \right\}.$$

Using the Ito’s formula for $\text{log}\;X_{i}(t)$, the system (2.2) can be written as

$$X_{i}(t)=X_{i}(0)\text{exp}\left\{\int_{0}^{t}f_{i}(s)ds-\left(\frac{1}{2}\sum_{j=1}^{N}\tau_{ij}^{2}\right)t+\sum_{j=1}^{N}\tau_{ij}W_{j}(t)\right\}\;\text{a.s.},$$

which means that if $X(0)\in R_{+}^{N}$, then $X(t)\in R_{+}^{N}$ a.s. for all $t\in \left[0,\tau_{e}\right)$. Due to (2.5), we can obtain the tightness of the family of transition probabilities of the solution X to the system (2.2). This means that $\tau_{e}=\infty$ a.s. Thus, for any initial value X(0) in $R_{+}^{N}$, the system (2.2) has a unique a.s. continuous strong solution X(t) in $R_{+}^{N}$ for all t∈[0,∞).

The positive definiteness of the matrix Σ in Assumption 3 ensures that the solution to the system (2.2) is nondegenerate. This is an important assumption because it makes sure that we have enough noise that can locally push the dynamics of the solution in all directions, and hence any positive solution state can move close to any other positive solution state in a finite time. In other words, this condition guarantees that the family of transition probabilities of the solution has a smooth density, which will be used for the proof of the persistence and extinction of the system (2.2).

Now, we define what we mean by persistence and extinction in our stochastic model.

**Definition 2.3.** *The process* X *is strongly stochastically persistent if it possesses a unique invariant probability measure* $\pi^{*}$ *in* $R_{+}^{N,\circ }$ *and*

$$\underset{t\to \infty }{\text{lim}}{\left\|P(t,x,\cdot )-\pi^{*}(\cdot )\right\|}_{TV}=0,\;x\in R_{+}^{N,\circ },$$

*where* $\|\cdot {\|}_{TV}$ *is the total variation norm and* P(t,x,·) *is the transition probability of* X.

**Definition 2.4.** If $X(0)=x\in R_{+}^{N,\circ }$, *we say that the species $X_{i}$ goes extinct with probability* $p_{x}>0$ *if*

$$P_{x}\left\{\underset{t\to \infty }{\text{lim}}X_{i}(t)=0\right\}=p_{x}.$$

*We say that the species* $X_{i}$ *goes extinct if, for all* $x\in R_{+}^{N,\circ }$,

$$P_{x}\left\{\underset{t\to \infty }{\text{lim}}X_{i}(t)=0\right\}=1.$$

In a stochastic setting, to look into the persistence and extinction, we consider the set ℳ as the collection of all ergodic invariant probability measures of the solution X(t) on the boundary $\partial R_{+}^{N}$. Clearly, ℳ≠∅, since $\delta^{*}\in ℳ$ is the Dirac measure concentrated at $0=(0,\cdots ,0{)}^{T}\in R_{+}^{N}$. For $\tilde{ℳ}\subseteq ℳ$, we use $\text{Conv}(\tilde{ℳ})$ to denote the convex hull of $\tilde{ℳ}$, which is the collection of probability measures of the form

$$\pi (\cdot )=\sum_{\mu \in \tilde{ℳ}}p_{\mu }\mu (\cdot )$$

with $p_{\mu }>0$ and $\sum_{\mu \in \tilde{ℳ}}p_{\mu }=1$. For $\delta^{*}\neq \mu \in ℳ$, since the stochastic system (2.2) is nondegenerate by Assumption 3, $1\leq n_{1}<\cdots <n_{k}\leq N$ exist such that the support of the ergodic invariant probability measure μ, denoted by supp(μ), is exactly $R_{+}^{\mu }$, in which

$$R_{+}^{\mu }≔\left\{\left(x_{1},\cdots ,x_{N}\right)\in R_{+}^{N}:x_{i}=0\;\text{for}\;i\in I_{\mu }^{c}\right\}$$

for $I_{\mu }≔\left\{n_{1},\cdots ,n_{k}\right\}$ and $I_{\mu }^{c}≔\{1,\cdots ,N\}\setminus I_{\mu }$. Let

$$R_{+}^{\mu ,\circ }≔\left\{\left(x_{1},\cdots ,x_{N}\right)\in R_{+}^{N}:x_{i}=0\;\text{for}\;i\in I_{\mu }^{c}\;\text{and}\;x_{i}>0\;\text{for}\;i\in I_{\mu }\right\}$$

and $\partial R_{+}^{\mu }≔R_{+}^{\mu }\setminus R_{+}^{\mu ,\circ }$. Similarly to a deterministic setting, persistence in a stochastic setting means that every ergodic invariant probability measure on the boundary is *a repeller*. Therefore, ergodic invariant probability measures play a similar role as equilibria in a deterministic setting. To determine an ergodic invariant probability measure of the stochastic system (2.3) to be a repeller, we compute its Lyapunov exponents by looking at the equations for $\text{ln}\;X_{i}(t),i=1,\cdots ,N$. Applying Ito’s formula for $\text{ln}\;X_{i}(t)$ gives

$$\frac{\text{ln}\;X_{i}(t)}{t}=\frac{\text{ln}\;X_{i}(0)}{t}+\frac{1}{t}\int_{0}^{t}f_{i}\left(X\left(s\right)\right)ds-\frac{1}{2}\sum_{j=1}^{N}\tau_{ij}^{2}+\sum_{j=1}^{N}\tau_{ij}\frac{W_{j}\left(t\right)}{t}.$$

When the solution X(t) to the system (2.3) is close to the support of an ergodic invariant probability measure μ,supp(μ), for a long time, then the Lyapunov exponent of the solution component $X_{i}$ with respect to μ (which is also called the *invasion rate* of species $X_{i}$ with respect to μ)

$$\lambda_{i}(\mu )≔\underset{t\to \infty }{\text{lim}}\frac{\text{ln}\;X_{i}(t)}{t}=\underset{t\to \infty }{\text{lim}}\frac{1}{t}\int_{0}^{t}f_{i}(X(s))ds-\frac{1}{2}\sum_{j=1}^{N}\tau_{ij}^{2},$$

can be approximated by the average with respect to μ (using the strong law of large numbers)

$$\int_{\partial R_{+}^{N}}f_{i}\left(x\right)\mu \left(dx\right)-\frac{1}{2}\sum_{j=1}^{N}\tau_{ij}^{2},$$

while the term

$$\frac{\text{ln}\;X_{i}(0)}{t}+\sum_{j=1}^{N}\tau_{ij}\frac{W_{j}(t)}{t},$$

is negligible. This implies that the Lyapunov exponents $\lambda_{i}(\mu ),i=1,\cdots ,N$, give the long-term growth rate of species $X_{i}$ if X(t) is close to supp(μ). Therefore, an ergodic invariant probability measure μ is a repeller if $\underset{i=1,\cdots ,N}{\text{max}}\lambda_{i}(\mu )>0$. Now we impose the following assumption that ensures the strong stochastic persistence.

**Assumption 4.** *For any* μ *in* Conv(ℳ),

$$\underset{i=1,\cdots ,N}{\text{max}}\lambda_{i}(\mu )>0,$$

*where*

$$\lambda_{i}\left(\mu \right)=\int_{\partial R_{+}^{N}}f_{i}\left(x\right)\mu \left(dx\right)-\frac{1}{2}\sum_{j=1}^{N}\tau_{ij}^{2},$$

**Theorem 2.3.** *Suppose that Assumptions 3 and 4 are true. Then the solution* X(t) *to the system* (2.2) *is strongly stochastically persistent, and its transition probabilities converge weakly to its unique invariant probability measure* $\pi^{*}$ in $R_{+}^{N,\circ }$ *exponentially fast in the total variation norm*.

Next, we make an assumption that will imply the extinction of the stochastic system.

**Assumption 5.** *There is a* μ∈ℳ *such that*

(2.6) $$\underset{i\in I_{\mu }^{c}}{\text{max}\;}\lambda_{i}(\mu )<0,$$

*If* $R_{+}^{\mu }\neq \{0\}$, *suppose further that for any* $v\in \text{Conv}\left({ℳ}_{\mu }\right)$, *we get*

(2.7) $$\underset{i\in I_{\mu }}{\text{max}\;}\lambda_{i}(v)>0,$$

*where* ${ℳ}_{\mu }≔\left\{v^{\prime }\in ℳ:\text{supp}\left(v^{\prime }\right)\subseteq \partial R_{+}^{\mu }\right\}$.

**Theorem 2.4.** *Under Assumptions 3 and 5, for any* δ>0 *sufficiently small and for any initial value* $x\in R_{+}^{N,\circ }$, *we obtain*

$$\underset{t\to \infty }{\text{lim}}E_{x}{\left(\overset{N}{\underset{i=1}{⋀}}X_{i}(t)\right)}^{\delta }=0.$$

We have several remarks for Assumption 5. First, if μ∈ℳ and $i\in I_{\mu }$, then $\lambda_{i}(\mu )=0$. Intuitively, this is because if the solution is inside the support of an ergodic invariant probability measure μ, then it is at an “equilibrium” and it does not tend to grow or decay. If μ∈ℳ and $\underset{i\in I_{\mu }^{c}}{\text{max}}\;\lambda_{i}(\mu )<0$, then μ is regarded as an “attractor”, which attracts solutions starting nearby. So we require the condition (2.6) to make sure that the solution component $X_{i}(t),i\in I_{\mu }^{c}$, will get close to 0 if it starts near $R_{+}^{\mu ,\circ }$. Second, the condition (2.7) is needed to guarantee that μ is really a “sink” in $R_{+}^{\mu ,\circ }$ but not the other invariant probability measures on $\partial R_{+}^{\mu }$, that is, if X is close to $R_{+}^{\mu ,\circ }$, then it is not pulled away to the boundary $\partial R_{+}^{\mu }$.

Finally, let

$${ℳ}^{1}≔\{\mu \in ℳ:\mu \;\text{satisfies Assumption}\;5\}$$

and ${ℳ}^{2}≔ℳ\setminus {ℳ}^{1}$. If there is no persistence, we would like to know exactly which species go extinct and which species survive. On the basis of the repulsion of the invariant probability measures in ${ℳ}^{2}$, we can derive the “attracting” region of invariant probability measures in ${ℳ}^{1}$ for the solution X. We make an additional assumption that ensures all the invariant probability measures outside $\text{Conv}\left({ℳ}^{1}\right)$ are repellers.

**Assumption 6.** *Suppose that one of the following holds true*:

- ${ℳ}^{2}=\emptyset$;
- *For any* $v\in \text{Conv}\left({ℳ}^{2}\right),\underset{i=1,\cdots ,N}{\text{max}}\;\lambda_{i}(v)>0$.

For any initial value $X(0)=x\in R_{+}^{N,\circ }$, let 𝒰=𝒰(ω) denote the weak*-limit set of the family of random occupation measures $\left\{{\tilde{\Pi }}_{t}(\cdot ),t\geq 0\right\}$ where

$${\tilde{\Pi }}_{t}\left(A\right)=\frac{1}{t}\int_{0}^{t}1_{\left\{X\left(s\right)\in A\right\}}ds,\;t>0,\;A\in ℬ\left(R_{+}^{N,\circ }\right),$$

which is the proportion of time the solution spends in a set A up to time t.

**Theorem 2.5.** *Suppose that Assumptions 1, 5, and 6 hold true and ${ℳ}^{1}\neq \emptyset$*. *Then for any $x\in R_{+}^{N,\circ }$*, *we obtain*

$$\sum_{\mu \in {ℳ}^{1}}P_{x}^{\mu }=1$$

*in which*

$$P_{x}^{\mu }≔P_{x}\left\{𝒰(\omega )=\{\mu \}\;\text{and}\;\underset{t\to \infty }{\text{lim}}\frac{\text{ln}\;X_{i}(t)}{t}=\lambda_{i}(\mu )<0,\;i\in I_{\mu }^{c}\right\},\;\mu \in {ℳ}^{1}.$$

### 2.3. Linearization method

Let us get started with the linearization of the deterministic system (2.3). We assume that all the equilibria of this system on the boundary $\partial R_{+}^{N,\circ }$ are unstable. Therefore, by Theorem 2.1, the unique positive equilibrium $E^{*}={\left(X_{1}^{*},\cdots ,X_{N}^{*}\right)}^{T}$ is locally stable. To understand the behavior of the deterministic system near $E^{*}$, we approximate the system (2.3) by the following linear system, as the solution is close to $E^{*}$:

(2.8) $$\frac{dX_{i}}{dt}=\phi_{0}^{i}+\sum_{j=1}^{N}\theta_{ij}\left(X_{j}-X_{j}^{*}\right),\;i=1,\cdots ,N,$$

where the values of $\phi_{0}^{i}$ and the $\theta_{ij}$ are constants to be determined. In fact, we would like to replace each right-hand side of the system (2.3), $h_{i}(X)$, by its linearized form $\phi_{0}^{i}+\sum_{j=1}^{N}\theta_{ij}\left(X_{j}-X_{j}^{*}\right)$ for i=1,⋯,N when $X_{1}-X_{1}^{*},\cdots ,X_{N}-X_{N}^{*}$ are close to 0. The constant coefficients $\phi_{0}^{i}$ and $\theta_{ij}$ are determined so that all the differences

$$\left|h_{i}(X)-\phi_{0}^{i}-\sum_{j=1}^{N}\theta_{ij}\left(X_{j}-X_{j}^{*}\right)\right|,$$

for i=1,⋯,N approximate 0 as X is close to $E^{*}$. If we use the norm $\|x{\|}_{2}=\sqrt{x_{1}^{2}+\cdots +x_{N}^{2}}$ in $R^{N}$ to identify how close two vectors are, then the linearization of the system (2.3) at the unique positive equilibrium $E^{*}$ is equivalent to finding the vector $\phi_{0}={\left(\phi_{0}^{1},\cdots ,\phi_{0}^{N}\right)}^{T}$ and the matrix $\Theta ={\left(\theta_{ij}\right)}_{N\times N}$ so that the difference

$${\left\|h(X)-\phi_{0}-\Theta \left(X-E^{*}\right)\right\|}_{2},$$

approximates 0 as ${\left\|X-E^{*}\right\|}_{2}\to 0$. For each i=1,⋯,N, applying the Taylor expansion for the function $h_{i}(X)$ at $E^{*}$ gives

$$h_{i}(X)=h_{i}\left(E^{*}\right)+\frac{\partial h_{i}\left(E^{*}\right)}{\partial X}\cdot \left(X-E^{*}\right)+\frac{1}{2}{\left(X-E^{*}\right)}^{T}\frac{\partial^{2}h_{i}\left(E^{*}\right)}{\partial X^{2}}\left(X-E^{*}\right)+\circ \left({\left\|X-E^{*}\right\|}_{2}^{2}\right),$$

in which

$$\frac{\partial h_{i}\left(E^{*}\right)}{\partial X}={\left(\frac{\partial h_{i}\left(E^{*}\right)}{\partial X_{1}},\cdots ,\frac{\partial h_{i}\left(E^{*}\right)}{\partial X_{N}}\right)}^{T}$$

and

$$\frac{\partial^{2}h_{i}\left(E^{*}\right)}{\partial X^{2}}={\left(\frac{\partial^{2}h_{i}\left(E^{*}\right)}{\partial X_{j}\partial X_{k}}\right)}_{N\times N}.$$

This implies that as ${\left\|X-E^{*}\right\|}_{2}\to 0$, we get

$$\left|h_{i}(X)-h_{i}\left(E^{*}\right)-\frac{\partial h_{i}\left(E^{*}\right)}{\partial X}\cdot \left(X-E^{*}\right)\right|\to 0,$$

Notice that $h_{i}\left(E^{*}\right)=0$ for each i=1,⋯,N. Therefore, when we choose $\phi_{0}=0$ and $\Theta =\frac{\partial h\left(E^{*}\right)}{\partial X}≔{\left(\frac{\partial h_{i}\left(E^{*}\right)}{\partial X_{j}}\right)}_{N\times N}$, then

$$h_{i}\left(X\right)\approx \sum_{j=1}^{N}\theta_{ij}\left(X_{j}-X_{j}^{*}\right),$$

as X is close to $E^{*}$. Then the linearization of the deterministic system (2.3) at $E^{*}$ takes the form

(2.9) $$\frac{dX_{i}}{dt}=\sum_{j=1}^{N}\frac{\partial h_{i}\left(E^{*}\right)}{\partial X_{j}}\left(X_{j}-X_{j}^{*}\right),\;i=1,\cdots ,N,$$

Next, we continue with the linearization of the stochastic system (2.2). We assume that the sufficient conditions (i.e., Assumption 3 and Assumption 4) for the strong persistence of the stochastic system are satisfied. By Theorem 2.3, there is a unique exponentially ergodic invariant probability measure $\pi^{*}$ in $R_{+}^{N,\circ }$. So, for any $x\in R_{+}^{N,\circ }$, all transition probabilities $P(t,x,\cdot )=P_{x}\{X(t)\in \cdot \}$ of the solution X(t) starting at x become the same and equal to $\pi^{*}$ as the time t becomes large enough. We call $\pi^{*}$ the *unique positive stationary solution* of the stochastic system (2.2). We say that the solution X(t) is close to $\pi^{*}$ if ${\left\|P(t,x,\cdot )-\pi^{*}(\cdot )\right\|}_{TV}$ is close to 0. Note that $E_{x}\pi^{*}$ approaches $E^{*}$ for any $x\in R_{+}^{N,\circ }$ when all entries in the noise intensity matrix Γ approach 0. Since, as time passes, all solutions X(t) will eventually end up in the unique positive stationary distribution no matter where it starts, we will drop the subscript x in the notation of $E_{x}$ and $P_{x}$ in this subsection.

The linearization of the stochastic system (2.2) at the stationary solution $\pi^{*}$ is to approximate this system by the following linear SDE system when the solution is close to $\pi^{*}$:

(2.10) $$dX_{i}=\left[\Phi_{i}+\sum_{j=1}^{N}\gamma_{ij}\left(X_{j}-m_{j}\right)\right]dt+X_{i}\sum_{j=1}^{N}\tau_{ij}dW_{j},\;i=1,\cdots ,N,$$

where $\left(m_{1},\cdots ,m_{N}\right)=E\pi^{*}$. In fact, our statistical linearization method is the replacement of each drift term $h_{i}(X)=X_{i}\left[\alpha_{i}+\sum_{j=1}^{N}\beta_{ij}X_{j}\right]$ in (2.2) by its linearized form $\Phi_{i}+\sum_{j=1}^{N}\gamma_{ij}\left(X_{j}-m_{j}\right)$ for i=1,⋯,N, in which the coefficients $\Phi_{i}$’s and $\gamma_{ij}$’s are determined by using the Taylor expansion of each $h_{i}(X)$ at $m=\left(m_{1},\cdots ,m_{N}\right)$. In other words, we would like to find these coefficients to minimize

(2.11) $$\left|h_{i}(X)-\Phi_{i}-\sum_{j=1}^{N}\gamma_{ij}\left(X_{j}-m_{j}\right)\right|,$$

as the solution X of the stochastic system (2.2) is close to $\pi^{*}$. As in the deterministic setting, we obtain

(2.12) $$\Phi_{i}=h_{i}(m)=m_{i}\left[\alpha_{i}+\sum_{j=1}^{N}\beta_{ij}m_{j}\right]\;i=1,\cdots ,N,$$

and for i,j=1,⋯,N

(2.13) $$\gamma_{ij}=\frac{\partial h_{i}}{\partial x_{j}}(m)=\left\{\begin{array}{ll} \alpha_{i}+\sum_{k=1}^{N}\beta_{ik}m_{k} & \text{if}\;i=j,\\ \beta_{ij}m_{i} & \text{if}\;i\neq j, \end{array}\right.$$

in which each $m_{i}$ can be computed using the strong law of large numbers

(2.14) $$m_{i}=\int_{\partial R_{+}^{N}}X_{i}\pi^{*}\left(dX\right)=\underset{t\to \infty }{\text{lim}}\frac{1}{t}\int_{0}^{t}X_{i}\left(s\right)ds,$$

Therefore the system (2.2) can be approximated by the linear SDE system at the positive stationary solution $\pi^{*}$

(2.15) $$d\left[\begin{array}{c} {\tilde{X}}_{1}\\ \cdots \\ {\tilde{X}}_{N} \end{array}\right]=\left(\left[\begin{array}{c} \Phi_{1}\\ \cdots \\ \Phi_{N} \end{array}\right]+\left[\begin{array}{ccc} \gamma_{11} & \cdots & \gamma_{N1}\\ \vdots & \ddots & \vdots \\ \gamma_{1N} & \cdots & \gamma_{NN} \end{array}\right]\left[\begin{array}{c} {\tilde{X}}_{1}-m_{1}\\ \cdots \\ {\tilde{X}}_{N}-m_{N} \end{array}\right]\right)dt+\sum_{i=1}^{N}\left[\begin{array}{ccc} \tau_{1i} & \cdots & 0\\ \vdots & \ddots & \vdots \\ 0 & \cdots & \tau_{Ni,} \end{array}\right]\left[\begin{array}{c} {\tilde{X}}_{1}\\ \cdots \\ {\tilde{X}}_{N} \end{array}\right]dW_{i}.$$

Finally, we comment on our linearization method. We know that the solution of the nonlinear stochastic system (2.2) is a Markov process X=X(t). In fact, our linearization is to find another Markov process, say $\tilde{X}=\tilde{X}(t)$, which represents the solution of the linear SDE system (2.15) such that both X and $\tilde{X}$ have the same mean, and the variance $E\|X-\tilde{X}{\|}_{2}^{2}$ is to be minimized as the time becomes large enough. In other words, when time goes by and becomes sufficiently large, the long-term behaviors of the two Markov processes X and $\tilde{X}$ are approximately the same. Because of that, a δ>0 exists such that the linear SDE system (2.15) is considered as a “ δ-perturbation” of the non-linear SDE system (2.2), and thus strong persistence of the system (2.2) implies that of the system (2.15) (see the definition of “ δ-perturbation” and Proposition 3 in [28] for more details).

### 2.4. Four resilience measures

On the basis of two excellent works by Arnoldi [19, 20], we indirectly establish four resilience measures, which are the *instantaneous return rate, average return rate, asymptotic resilience, and convergence rate* of our stochastic gLV model, through the second moment of the solutions of the corresponding linearized SDE system. These four resilience quantities, in which the first two are functions of time and the last two are just numbers, measure how strong the recovery dynamics of our original stochastic system is after a pulse perturbation is applied.

Now we start building up these four stability concepts by letting

$$A=\left[\begin{array}{ccc} \gamma_{11} & \cdots & \gamma_{N1}\\ \vdots & \ddots & \vdots \\ \gamma_{1N} & \cdots & \gamma_{NN} \end{array}\right],\;B_{i}=\left[\begin{array}{ccc} \tau_{1i} & \cdots & 0\\ \vdots & \ddots & \vdots \\ 0 & \cdots & \tau_{Ni} \end{array}\right],\;i=1,\cdots ,N,$$

$$\Theta ={\left(\Phi_{1},\cdots ,\Phi_{N}\right)}^{T},\;m={\left(m_{1},\cdots ,m_{N}\right)}^{T},\;\text{and}\;Y=\tilde{X}-m.$$

Then (2.15) can be written in matrix form:

(2.16) $$dY=\left(AY+\Theta \right)dt+\sum_{i=1}^{N}\left(B_{i}Y+B_{i}m\right)dW_{i}.$$

We assume that the linearized system (2.16) is already at its equilibrium state. A pulse perturbation at time t=0 applied to this linear SDE system is characterized by a vector u=Y(0), which describes the state of the system right after the perturbation. Then the recovery trajectory Y(t) after this pulse perturbation is given by

(2.17) $$Y(t)=\Phi (t)u+\Phi (t)\int_{0}^{t}\Phi^{-1}(s)\left[\Theta -\sum_{i=1}^{N}B_{i}^{2}m\right]ds+\sum_{i=1}^{N}\Phi (t)\int_{0}^{t}\Phi^{-1}(s)B_{i}mdW_{i}(s),$$

in which Φ(t) is the solution matrix of the homogeneous system

(2.18) $$dY=AYdt+\sum_{i=1}^{N}B_{i}YdW_{i}.$$

with the initial condition Φ(0)=I (the identity matrix of size N). In general, we cannot explicitly compute the solution Y(t) in terms of $A,B_{1},\ldots ,B_{N}$. Notice that the solution Y(t) is nowhere differentiable. Without the smoothness of the solution, it is difficult to build up stability concepts of a dynamic system that are able to be tractable. To avoid this difficulty, we can smooth out the solutions of the linearized system (2.16) by looking at the dynamics of their second moments. To proceed, let $P(t)=E\left(Y(t)Y(t{)}^{T}\right)$ be the expectation of the matrix-valued process $Y(t)Y(t{)}^{T}$, which is given by the unique solution of the matrix ODE (see [23])

(2.19) $$\frac{dP}{dt}=AP+PA^{T}+\sum_{i=1}^{N}B_{i}PB_{i}+\Theta M^{T}+M\Theta^{T}+\sum_{i=1}^{N}B_{i}\left(Mm^{T}+mM^{T}+{mm}^{T}\right)B_{i},$$

with the initial value $P(0)=Y(0)Y(0{)}^{T}={uu}^{T}$, and M≔M(t)=EY(t) is the solution to the linear ODE system, with the initial value M(0)=u,

(2.20) $$\dot{M}=AM+\Theta .$$

We can treat the matrix P(t) as a $N^{2}$-dimensional vector

$$Z(t)≔\text{vec}(P(t))={\left(Z_{1}(t),\cdots ,Z_{N^{2}}(t)\right)}^{T}\;=\left(E\left(Y_{1}^{2}(t)\right),E\left(Y_{2}(t)Y_{1}(t)\right),\cdots ,E\left(Y_{N}(t)Y_{1}(t)\right),{\left.E\left(Y_{1}(t)Y_{2}(t)\right),E\left(Y_{2}(t{)}^{2}\right),\cdots ,E\left(Y_{N}(t)Y_{2}(t)\right),\cdots ,E\left(Y_{N}(t{)}^{2}\right)\right)}^{T}.\right.$$

Applying the vectorization operation on both sides of the system (2.19) and then using the properties of Kronecker products and matrix vectorization in linear algebra [23], we obtain the equivalent $N^{2}$-dimensional ODE system

(2.21) $$\frac{dZ}{dt}=SZ+T,$$

in which

$$S=I⊗A+A⊗I+\sum_{i=1}^{N}B_{i}⊗B_{i},$$

and

$$T=\text{vec}\left(\Theta {\text{M}}^{\text{T}}+\text{M}\Theta^{\text{T}}\right)+\sum_{\text{i}=1}^{\text{N}}\left({\;\text{B}}_{\text{i}}⊗{\;\text{B}}_{\text{i}}\right)\text{vec}\left({\text{M}m}^{\text{T}}+{m\text{M}}^{\text{T}}+{mm}^{\text{T}}\right),$$

The matrix S is called the *mean-square stability matrix* of the linear SDE system (2.16). The stationary solution to (2.16) is globally mean-square asymptotically stable if $\alpha (S)={\text{max}}_{i=1,\cdots ,N^{2}}\;\text{Re}\left(\lambda_{i}\right)<0$, where $\text{Re}\left(\lambda_{i}\right)$ is the real part of the eigenvalue $\lambda_{i}$ of the matrix S. Now let

$$W\left(t\right)≔\sum_{i=1}^{N}EY_{i}^{2}\left(t\right)=E\left(\sum_{i=1}^{N}Y_{i}^{2}\left(t\right)\right)=E\left(Y^{T}\left(t\right)Y\left(t\right)\right),$$

then W(t) measures the average squared distance of the solution X(t) of the system (2.2) to the expectation of its equilibrium state $\pi^{*}$. We define four resilience quantities based on W(t) as follows:

${ℛ}_{t}^{\text{ins}}=-\frac{d}{dt}\;\text{ln}\;W(t{)}^{1/2}$ is the instantaneous return rate at time t>0,
${ℛ}_{t}^{\text{ave}}=-\frac{\text{ln}\;W(t{)}^{1/2}-\text{ln}\;W(0{)}^{1/2}}{t}$ is the average return rate over the time interval [0,t],
${ℛ}_{c}=-\frac{1}{2}\alpha (S)$ is the rate of convergence of the ODE system (2.21), and
${ℛ}_{\infty }=\underset{t\to \infty }{\text{lim}}{ℛ}_{t}^{\text{ave}}$ is the asymptotic resilience of the ODE system (2.21).

In these definitions, ${ℛ}_{t}^{\text{ins}}$ and ${ℛ}_{t}^{\text{ave}}$ are deterministic functions of time t, which capture the short-term behavior of the recovery trajectory Y(t), while ${ℛ}_{c}$ and ${ℛ}_{\infty }$ are only numbers, which represent the long-term dynamics of the solution’s recovery. These two functions and two numbers give us more tractable computations that measure the recovery dynamics of the stochastic system (2.16) since we are able to explicitly compute the quantity ln $W(t{)}^{1/2}$ and its derivative in terms of the matrices S and T. In fact, two quantities ${ℛ}_{t}^{\text{ins}}$ and ${ℛ}_{t}^{\text{ave}}$ can be computed on the basis of the derivative of lnW(t)

$$\frac{d}{dt}\;\text{ln}\;W=\frac{1}{W}\frac{dW}{dt}=\frac{1}{W}\left\{E\left(Y^{T}\left(A^{T}+A\right)Y\right)+\Theta^{T}M+M^{T}\Theta +\sum_{i=1}^{N}\left[E\left(Y^{T}B_{i}^{2}Y\right)+m^{T}B_{i}^{2}M+M^{T}B_{i}^{2}m+m^{T}B_{i}^{2}m\right]\right\}\;=\frac{1}{W}\left\{{\left[\text{vec}\left({\;\text{A}}^{\text{T}}+\text{A}\right)+\sum_{\text{i}=1}^{\text{N}}\text{vec}\left({\;\text{B}}_{\text{i}}^{2}\right)\right]}^{T}Z+\Theta^{T}M+M^{T}\Theta +\sum_{i=1}^{N}\left(m^{T}B_{i}^{2}M+M^{T}B_{i}^{2}m+m^{T}B_{i}^{2}m\right)\right\},$$

where W,M, and Z can be computed from two ODE systems (2.20) and (2.21). Lastly, when all noise intensities are equal to 0 in the system (2.2) (that is, $B_{i}=0$ for all i=1,⋯,N), all our definitions of the four resilience measures above correspond to those defined in Arnoldi [20] in which the rate of convergence and the asymptotic resilience coincide.

## 3. Example of a stochastic 2-species model

### 3.1. Analysis of the model

To demonstrate the method we proposed in previous section, let us look at the stochastic gLV model of two species

(3.1) $$dX_{1}=X_{1}\left(\alpha_{1}-\beta_{11}X_{1}+\beta_{12}X_{2}\right)dt+X_{1}\left(\tau_{11}dW_{1}+\tau_{12}dW_{2}\right),\;dX_{2}=X_{2}\left(\alpha_{2}+\beta_{21}X_{1}-\beta_{22}X_{2}\right)dt+X_{2}\left(\tau_{21}dW_{1}+\tau_{22}dW_{2}\right),$$

where $\beta_{11}>0$ and $\beta_{22}>0$. There are three cases:

- If $\beta_{12}<0$ and $\beta_{21}<0$, then the system (3.1) is a competitive model.
- If $\beta_{12}>0$ and $\beta_{21}>0$, then the system (3.1) is a mutualistic model.
- If $\left(\beta_{12}<0,\beta_{21}>0\right)$ or $\left(\beta_{12}>0,\beta_{21}<0\right)$, then the system (3.1) is a predator–prey model (mixed competition and mutualism).

Next, we analyze the dynamics of the system (3.1) on the boundary of $R_{+}^{2}$, which is $\partial R_{+}^{2}≔R_{1+}^{\circ }\cup R_{2+}^{\circ }\cup \{(0,0)\}$, where $R_{1+}^{\circ }≔\left\{\left(x_{1},0\right):x_{1}>0\right\}$ and $R_{2+}^{\circ }≔\left\{\left(0,x_{2}\right):x_{2}>0\right\}$.

Case 1. If $X_{1}(0)=0$ and $X_{2}(0)=0$, then, by (3.1), we get, for a.s.

$$X_{1}(t)=X_{1}(0)\text{exp}\left\{\int_{0}^{t}\left[\alpha_{1}-\beta_{11}X_{1}(s)+\beta_{12}X_{2}(s)-\frac{\tau_{11}^{2}}{2}-\frac{\tau_{12}^{2}}{2}\right]dt+\tau_{11}W_{1}(t)+\tau_{12}W_{2}(t)\right\},$$

$$X_{2}(t)=X_{2}(0)\text{exp}\left\{\int_{0}^{t}\left[\alpha_{2}+\beta_{21}X_{1}(s)-\beta_{22}X_{2}(s)-\frac{\tau_{21}^{2}}{2}-\frac{\tau_{22}^{2}}{2}\right]dt\left.+\tau_{21}W_{1}(t)+\tau_{22}W_{2}(t)\right\}.\right.$$

This implies that $X_{1}(t)=X_{2}(t)=0$ for all t>0 a.s. So, the Dirac delta measure at $(0,0),\mu_{0}$, is an ergodic invariant probability measure for the system (3.1) on the boundary $\partial R_{+}^{2}$.

Case 2. If $X_{1}(0)=0$ and $X_{2}(0)>0$, then, as in Case 1, $X_{1}(t)\equiv 0$ a.s. and $X_{2}(t)>0$ for all t>0 a.s. Plugging in 0 into $X_{1}$ in the second equation of (3.1) gives

(3.2) $$dX_{2}=X_{2}\left(\alpha_{2}-\beta_{22}X_{2}\right)dt+X_{2}\left(\tau_{21}dW_{1}+\tau_{22}dW_{2}\right).$$

For a fixed $\alpha_{2}>0$, consider

$$s\left(X_{2}\right)=\int_{\alpha_{2}}^{X_{2}}\text{exp}\;\left\{-\int_{\alpha_{2}}^{y}\frac{\left(2\alpha_{2}-2\beta_{22}u\right)u}{\left(\tau_{21}^{2}+\tau_{22}^{2}\right)u^{2}}du\right\}dy\;=C_{2}\int_{\alpha_{2}}^{X_{2}}y^{-\frac{2\alpha_{2}}{\tau_{21}^{2}+\tau_{22}^{2}}}\;\text{exp}\;\left\{\frac{2\beta_{22}}{\tau_{21}^{2}+\tau_{22}^{2}}y\right\}dy.$$

Since the integrand in the last integral can be written as

$$y^{\frac{-2\alpha_{2}}{\tau_{21}^{2}+\tau_{22}^{2}}}\left[1+\frac{2\beta_{22}}{\tau_{21}^{2}+\tau_{22}^{2}}y+\frac{1}{2!}\frac{4\beta_{22}^{2}}{{\left(\tau_{21}^{2}+\tau_{22}^{2}\right)}^{2}}y^{2}+\cdots \right],$$

a $k\in Z_{+}$ exists such that $\frac{-2\alpha_{2}}{\tau_{21}^{2}+\tau_{22}^{2}}+k>-1,s(\infty )=\infty$. If $\frac{-2\alpha_{2}}{\tau_{21}^{2}+\tau_{22}^{2}}+1>0$, which is equivalent to $\alpha_{2}-\frac{\tau_{21}^{2}}{2}-\frac{\tau_{22}^{2}}{2}<0$, then s(0+)>−∞. This means that $\underset{t\to \infty }{\text{lim}}X_{2}(t)=0$ a.s. and so the equation (3.2) does not have any invariant probability measure on $R_{+}^{\circ }$. If $\frac{-2\alpha_{2}}{\tau_{21}^{2}+\tau_{22}^{2}}+1<0$, which is equivalent to $\alpha_{2}-\frac{\tau_{21}^{2}}{2}-\frac{\tau_{22}^{2}}{2}>0$, then s(0+)=−∞. Therefore, $X_{2}(t)$ oscillates between 0 and ∞. Thus the equation (3.2) has a unique invariant probability measure $\pi_{2}$ on $R_{+}^{\circ }$, which is the Gamma distribution

$$\pi_{2}\sim \text{Gamma}\left(\frac{2\alpha_{2}}{\tau_{21}^{2}+\tau_{22}^{2}}-1,\frac{\tau_{21}^{2}+\tau_{22}^{2}}{2\beta_{22}}\right).$$

So $\mu_{2}≔\delta_{0}^{*}\times \pi_{2}$ is an ergodic invariant probability measure for the system (3.1) on the boundary $R_{2+}^{\circ }$, in which $\delta_{0}^{*}$ is the Dirac Delta measure with the mass at 0.

Case 3. If $X_{1}(0)>0$ and $X_{2}(0)=0$ then $X_{1}(t)>0$ for all t>0 a.s. and $X_{2}(t)\equiv 0$ a.s. But then the first equation of (3.1) becomes

(3.3) $$dX_{1}=X_{1}\left(\alpha_{1}-\beta_{11}X_{1}\right)dt+X_{1}\left(\tau_{11}dW_{1}+\tau_{12}dW_{2}\right).$$

By the same argument as above, the equation (3.3) has a unique invariant probability measure $\pi_{1}$ on $R_{+}^{\circ }$, which is the Gamma distribution

$$\pi_{1}\sim \text{Gamma}\left(\frac{2\alpha_{1}}{\tau_{11}^{2}+\tau_{12}^{2}}-1,\frac{\tau_{21}^{2}+\tau_{22}^{2}}{2\beta_{11}}\right),$$

provided that $\alpha_{1}-\frac{\tau_{11}^{2}}{2}-\frac{\tau_{12}^{2}}{2}>0$. Otherwise, (3.3) does not have any invariant probability measure on $R_{+}^{\circ }$. Therefore, $\mu_{1}=\pi_{1}\times \delta_{0}^{*}$ is an ergodic invariant probability measure for the system (3.1) on the boundary $R_{1+}^{\circ }$.

Now we compute the Lyapunov exponents of $\mu_{0}=\delta_{0}^{*}\times \delta_{0}^{*},\mu_{1}$, and $\mu_{2}$. By Ito’s formula, the system (3.1) is equivalent to

$$\frac{\text{ln}\;X_{1}(t)}{t}=\frac{\text{ln}\;X_{1}(0)}{t}+\frac{1}{t}\int_{0}^{t}\left[\alpha_{1}-\beta_{11}X_{1}(s)+\beta_{12}X_{2}(s)-\frac{\tau_{11}^{2}}{2}-\frac{\tau_{12}^{2}}{2}\right]ds+\tau_{11}\frac{W_{1}(t)}{t}+\tau_{12}\frac{W_{2}(t)}{t},$$

$$\frac{\text{ln}\;X_{2}(t)}{t}=\frac{\text{ln}\;X_{2}(0)}{t}+\frac{1}{t}\int_{0}^{t}\left[\alpha_{2}+\beta_{21}X_{1}(s)-\beta_{22}X_{2}(s)-\frac{\tau_{21}^{2}}{2}-\frac{\tau_{22}^{2}}{2}\right]ds+\tau_{21}\frac{W_{1}(t)}{t}+\tau_{22}\frac{W_{2}(t)}{t}.$$

By the strong law of large numbers, when the solution $X(t)={\left(X_{1}(t),X_{2}(t)\right)}^{T}$ of (3.1) is close to $\text{supp}\left(\delta^{*}\right)=\{(0,0)\}$ for a long time (meaning that the solution X(t) is within some small neighborhood of (0, 0) for a long time), the Lyapunov exponent for the first component (the invasion rate of Species 1) with respect to $\mu_{0}$ is

$$\lambda_{1}\left(\mu_{0}\right)=\underset{t\to \infty }{\text{lim}}\frac{\text{ln}\;X_{1}\left(t\right)}{t}\;=\underset{t\to \infty }{\text{lim}\;}\frac{1}{t}\;\int_{0}^{t}\left[\alpha_{1}-\beta_{11}X_{1}\left(s\right)+\beta_{12}X_{2}\left(s\right)-\frac{\tau_{11}^{2}}{2}-\frac{\tau_{12}^{2}}{2}\right]ds\;=\int_{\partial {\mathbb{R}}_{+}^{2}}\left[\alpha_{1}-\beta_{11}X_{1}+\beta_{12}X_{2}-\frac{\tau_{11}^{2}}{2}-\frac{\tau_{12}^{2}}{2}\right]\mu_{0}\left(dX\right)\;=\alpha_{1}-\frac{\tau_{11}^{2}}{2}-\frac{\tau_{12}^{2}}{2}.$$

Similarly,

$$\lambda_{2}\left(\mu_{0}\right)=\int_{\partial R_{+}^{2}}\left[\alpha_{2}+\beta_{21}X_{1}+\beta_{22}X_{2}-\frac{\tau_{21}^{2}}{2}-\frac{\tau_{22}^{2}}{2}\right]\mu_{0}(dX)\;=\alpha_{2}-\frac{\tau_{21}^{2}}{2}-\frac{\tau_{22}^{2}}{2}.$$

By the similar computations, the Lyapunov exponents of $\mu_{1}$ and $\mu_{2}$ can be computed as

$$\lambda_{1}\left(\mu_{1}\right)=0,$$

$$\lambda_{2}\left(\mu_{1}\right)=\alpha_{2}-\frac{\tau_{21}^{2}}{2}-\frac{\tau_{22}^{2}}{2}+\frac{\beta_{21}}{\beta_{11}}\alpha_{1}-\frac{\beta_{21}}{2\beta_{11}}\left(\tau_{11}^{2}+\tau_{12}^{2}\right),$$

$$\lambda_{1}\left(\mu_{2}\right)=\alpha_{1}-\frac{\tau_{11}^{2}}{2}-\frac{\tau_{12}^{2}}{2}+\frac{\beta_{12}}{\beta_{22}}\alpha_{2}-\frac{\beta_{12}}{2\beta_{22}}\left(\tau_{21}^{2}+\tau_{22}^{2}\right),$$

$$\lambda_{2}\left(\mu_{2}\right)=0.$$

On the basis of the Lyapunov exponents of the set of all ergodic invariant probability measures of the system (3.1) on the boundary $\partial R_{+}^{2}$, denoted $ℳ=\left\{\mu_{0},\mu_{1},\mu_{2}\right\}$, we can give the complete classification of the dynamics of the system (3.1). First of all, we assume that the matrix

$$\Sigma =\left[\begin{array}{ll} \sigma_{11} & \sigma_{12}\\ \sigma_{21} & \sigma_{22} \end{array}\right],$$

where $\sigma_{ij}=\tau_{i1}\tau_{j1}+\tau_{i2}\tau_{j2}(i,j=1,2)$, is positive definite. This is equivalent to requiring that

$$\tau_{11}^{2}+\tau_{12}^{2}>0,\tau_{21}^{2}+\tau_{22}^{2}>0,\;\text{and}\;\tau_{11}\tau_{22}-\tau_{12}\tau_{21}\neq 0.$$

Next, we claim that the family of transition probability functions of the solution to the system (3.1) is tight when the system (3.1) is either competitive or predator-prey. Furthermore, when the system (3.1) is mutualistic, the family of transition probability functions of its solution might not be tight. In this case, we show that the solution blows up in finite time almost surely or that there is no invariant measure on $R_{+}^{2,\circ }$. Indeed, we first assume $\beta_{12}<0$ and $\beta_{21}<0$. In order to prove the tightness of the solution of (3.1), it suffices to show that there is a δ>0 such that

(3.4) $$\frac{X_{1}f_{1}(X)+X_{2}f_{2}(X)}{1+X_{1}+X_{2}}-\frac{\sum_{i,j=1}^{2}\sigma_{ij}X_{i}X_{j}}{2{\left(1+X_{1}+X_{2}\right)}^{2}}+\delta \left(3+\left|f_{1}(X)\right|+\left|f_{2}(X)\right|\right)<0$$

for any $\|X\|=\left|X_{1}\right|+\left|X_{2}\right|$ that is sufficiently large, where $f_{1}(X)=X_{1}\left(\alpha_{1}-\beta_{11}X_{1}+\beta_{12}X_{2}\right)$ and $f_{2}(X)=X_{2}\left(\alpha_{2}+\beta_{21}X_{1}-\beta_{22}X_{2}\right)$. Since $\beta_{12}$ and $\beta_{21}$ are both negative, as ‖X‖→∞, we get

$$\frac{X_{1}f_{1}(X)+X_{2}f_{2}(X)}{{\left(1+X_{1}+X_{2}\right)}^{2}}=\frac{\alpha_{1}X_{1}+\alpha_{2}X_{2}-\left(\beta_{11}X_{1}^{2}+\beta_{22}X_{2}^{2}\right)+\left(\beta_{12}+\beta_{21}\right)X_{1}X_{2}}{{\left(1+X_{1}+X_{2}\right)}^{2}}$$

which approaches −∞. So there is a $\tilde{\beta }>0$ such that for any ‖X‖ that is sufficiently large

$$\frac{X_{1}f_{1}(X)+X_{2}f_{2}(X)}{1+X_{1}+X_{2}}<-\tilde{\beta }\left(1+X_{1}+X_{2}\right).$$

On the other hand, using the Cauchy–Schwarz’s inequality, a $\tilde{\sigma }>0$ exists such that

$$-\frac{\sum_{i,j=1}^{2}\sigma_{ij}X_{i}X_{j}}{2{\left(1+X_{1}+X_{2}\right)}^{2}}\leq -\tilde{\sigma }\frac{X_{1}^{2}+X_{2}^{2}}{{\left(1+X_{1}+X_{2}\right)}^{2}}\leq -\tilde{\sigma }$$

when ‖X‖ is sufficiently large. Then we can choose a δ>0 that is small enough so that we can obtain the inequality (3.4) as ‖X‖ is large enough. Second, we suppose either $\left(\beta_{12}<0,\beta_{21}>0\right)$ or $\left(\beta_{12}>0,\beta_{21}<0\right)$. Then $c_{1}>0$ and $c_{2}>0$ exist such that $c_{1}\beta_{12}+c_{2}\beta_{21}=0$. This implies that

$$\underset{\|X\|\to \infty }{\text{lim}}\frac{c_{1}X_{1}f_{1}(X)+c_{2}X_{2}f_{2}(X)}{{\left(1+c_{1}X_{1}+c_{2}X_{2}\right)}^{2}}=-\infty .$$

By the same reasoning as in the competitive case, we can easily find a $\delta^{\prime }>0$ that is sufficiently small such that, for any ‖X‖ large enough, we get the inequality (3.4). Lastly, for the case $\beta_{12}>0$ and $\beta_{21}>0$, we show that the solution of (3.1) blows up in finite time almost surely under the assumptions that

$$\alpha_{1}-\frac{\tau_{11}^{2}}{2}-\frac{\tau_{12}^{2}}{2}>0,\;\alpha_{2}-\frac{\tau_{21}^{2}}{2}-\frac{\tau_{22}^{2}}{2}>0,\;\text{and}\;\beta_{11}\beta_{22}-\beta_{12}\beta_{21}\leq 0.$$

By way of contradiction, assume that X(t) does not blow up in finite time and has an invariant probability measure on $R_{+}^{2,\circ }$. As a result, X(t) is a recurrent process. But then, by Ito’s formula,

$$\frac{\beta_{22}\;\text{ln}\;X_{1}(t)+\beta_{12}\;\text{ln}\;X_{2}(t)}{t}=\frac{\beta_{22}\;\text{ln}\;X_{1}(0)+\beta_{12}\;\text{ln}\;X_{2}(0)}{t}\;+\beta_{22}\left(\alpha_{1}-\frac{\tau_{11}^{2}}{2}-\frac{\tau_{12}^{2}}{2}\right)+\beta_{12}\left(\alpha_{2}-\frac{\tau_{21}^{2}}{2}-\frac{\tau_{22}^{2}}{2}\right)\;+\left(\beta_{12}\beta_{21}-\beta_{11}\beta_{22}\right)\frac{1}{t}\int_{0}^{t}X_{1}\left(s\right)ds\;+\left(\tau_{1}1\beta_{22}+\tau_{21}\beta_{12}\right)\frac{W_{1}\left(t\right)}{t}\;+\left(\tau_{12}\beta_{22}+\tau_{22}\beta_{12}\right)\frac{W_{2}\left(t\right)}{t}.$$

It follows that, by the above assumptions,

$$\underset{t\to \infty }{\text{lim}\;\text{sup}}\frac{\beta_{22}\;\text{ln}\;X_{1}(t)+\beta_{12}\;\text{ln}\;X_{2}(t)}{t}>0\;\text{a.s.}$$

which is a contradiction. Therefore, the system (3.1), which is competitive or predator-prey, satisfies Assumption 3 in Subsection 2.2.

Now we focus on the stochastic model (3.1) of the competitive type and predator–prey type. On the basis of Theorems 2.3, 2.4, and 2.5 in Subsection 2.2, we are able to describe the complete dynamic picture of the system (3.1) as follows.

**C1.** Suppose $\lambda_{1}\left(\mu_{0}\right)<0$ and $\lambda_{2}\left(\mu_{0}\right)<0$. This means that there are no other ergodic invariant probability measures in $\partial R_{+}^{2}$ in addition to $\mu_{0}$. Then $ℳ=\left\{\mu_{0}\right\}$, which implies that Assumptions 3 and 5 hold for the system (3.1). By Theorem 2.4, the solution $X(t)={\left(X_{1}(t),X_{2}(t)\right)}^{T}$ converges a.s. to $(0,0{)}^{T}$ for any initial condition $x=\left(x_{1},x_{2}\right)\in R_{+}^{2,\circ }$.

**C2.** Suppose $\lambda_{1}\left(\mu_{0}\right)>0$ and $\lambda_{2}\left(\mu_{0}\right)<0$. Then $ℳ=\left\{\mu_{0},\mu_{1}\right\}$. There are two cases.

- If $\lambda_{2}\left(\mu_{1}\right)>0$ then Assumption 4 is fulfilled and hence transition probability function of the solution to the system (3.1) converges to an invariant probability measure ${\overset{‾}{\mu }}_{1}$ in $R_{+}^{2,\circ }$ in total variation norm.
- If $\lambda_{2}\left(\mu_{1}\right)<0$ then ${ℳ}^{1}=\left\{\mu_{1}\right\}$ and so ${ℳ}^{2}=ℳ\setminus {ℳ}^{1}=\left\{\mu_{0}\right\}$. This means that Assumptions 3 and 6 are satisfied. By Theorem 2.5, $X_{2}(t)$ converges to 0 with the exponential rate $\lambda_{2}\left(\mu_{1}\right)$ and the family of random occupation measures of the solution converges to $\mu_{1}$.

**C3.** Suppose $\lambda_{1}\left(\mu_{0}\right)<0$ and $\lambda_{2}\left(\mu_{0}\right)>0$. Then $ℳ=\left\{\mu_{0},\mu_{2}\right\}$. There are 2 cases:

- If $\lambda_{1}\left(\mu_{2}\right)>0$, then Assumption 4 is fulfilled, and hence the transition probability function of the solution to the system (3.1) converges to an invariant probability measure ${\overset{‾}{\mu }}_{2}$ in $R_{+}^{2,\circ }$ in the total variation norm.
- If $\lambda_{1}\left(\mu_{2}\right)<0$, then ${ℳ}^{1}=\left\{\mu_{2}\right\}$ and so ${ℳ}^{2}=ℳ\setminus {ℳ}^{1}=\left\{\mu_{0}\right\}$. This means that Assumptions 3 and 6 are satisfied. By Theorem 2.5, $X_{1}(t)$ converges to 0 with the exponential rate $\lambda_{1}\left(\mu_{2}\right)$, and the family of random occupation measures of the solution converges to $\mu_{2}$.

**C4.** Suppose $\lambda_{1}\left(\mu_{0}\right)>0$ and $\lambda_{2}\left(\mu_{0}\right)>0$. Then $ℳ=\left\{\mu_{0},\mu_{1},\mu_{2}\right\}$. There are four cases.

- If $\lambda_{1}\left(\mu_{2}\right)>0$ and $\lambda_{2}\left(\mu_{1}\right)>0$, then any invariant probability measure on $\partial R_{+}^{2}$ has the form $\mu =p_{0}\mu_{0}+p_{1}\mu_{1}+p_{2}\mu_{2}$, where $p_{0},p_{1}$, and $p_{2}$ are positive numbers such that $p_{0}+p_{1}+p_{2}=1$. It is straightforward that $\text{max}\left\{\lambda_{1}(\mu ),\lambda_{2}(\mu )\right\}>0$. Then Assumptions 3 and 4 are satisfied. By Theorem 2.3, there is a unique invariant probability measure $\mu^{*}$ on $R_{+}^{2,\circ }$, and the transition probability function $P(t,x,\cdot ),x\in R_{+}^{2,\circ }$, converges to $\mu^{*}$ in the total variation norm.
- If $\lambda_{1}\left(\mu_{2}\right)<0$ and $\lambda_{2}\left(\mu_{1}\right)<0$, then ${ℳ}^{1}=\left\{\mu_{1},\mu_{2}\right\}$ and so ${ℳ}^{2}=ℳ\setminus {ℳ}^{1}=\left\{\mu_{0}\right\}$. Hence, Assumptions 3 and 6 hold true. By Theorem 2.5, $p_{1}^{x}>0,p_{2}^{x}>0$, and $p_{1}^{x}+p_{2}^{x}=1$, where
$$p_{1}^{x}=P_{x}\left\{𝒰(\omega )=\left\{\mu_{1}\right\}\;\text{and}\;\underset{t\to \infty }{\text{lim}}\frac{\text{ln}\;X_{2}(t)}{t}=\lambda_{2}\left(\mu_{1}\right)<0\right\},$$
$$p_{2}^{x}=P_{x}\left\{𝒰(\omega )=\left\{\mu_{2}\right\}\;\text{and}\;\underset{t\to \infty }{\text{lim}}\frac{\text{ln}\;X_{1}(t)}{t}=\lambda_{1}\left(\mu_{2}\right)<0\right\}.$$
- If $\lambda_{1}\left(\mu_{2}\right)>0$ and $\lambda_{2}\left(\mu_{1}\right)<0$, then the conclusion is the same as in C2(ii).
- If $\lambda_{1}\left(\mu_{2}\right)<0$ and $\lambda_{2}\left(\mu_{1}\right)>0$, then the conclusion is the same as in C3(ii).

Finally, to study the resilience of the two-dimensional stochastic gLV model (3.1), we assume that the system (3.1) is competitive or predator-prey type with the matrix Σ being positive-definite and satisfies the inequality (3.4) for some δ>0. Furthermore, we assume that the system (3.1) satisfies the assumptions as in C2(i) or C3(i) or C4(i).

Then the stochastic gLV model of two species is stochastically strongly persistent, that is, it has a unique positive stationary distribution $\pi^{*}$ on $R_{+}^{2,\circ }$ such that the transition probability function of its solution converges to $\pi^{*}$ in the total variation norm. The linearized system of (3.1) at $\pi^{*}$ is given by

(3.5) $$d{\tilde{X}}_{1}=\left[\Phi_{1}+\gamma_{11}\left({\tilde{X}}_{1}-m_{1}\right)+\gamma_{12}\left({\tilde{X}}_{2}-m_{2}\right)\right]dt+{\tilde{X}}_{1}\left(\tau_{11}dW_{1}+\tau_{12}dW_{2}\right),\;d{\tilde{X}}_{2}=\left[\Phi_{2}+\gamma_{21}\left({\tilde{X}}_{1}-m_{1}\right)+\gamma_{22}\left({\tilde{X}}_{2}-m_{2}\right)\right]dt+{\tilde{X}}_{2}\left(\tau_{21}dW_{1}+\tau_{22}dW_{2}\right),$$

where the values of $\theta_{i}$ and the $\gamma_{ij}$ are determined by

$$\Phi_{1}=\alpha_{1}m_{1}-\beta_{11}m_{1}^{2}+\beta_{12}m_{1}m_{2},$$

$$\Phi_{2}=\alpha_{2}m_{2}+\beta_{21}m_{2}m_{1}-\beta_{22}m_{2}^{2},$$

$$\gamma_{11}=\alpha_{1}+2\beta_{11}m_{1}+\beta_{12}m_{2},$$

$$\gamma_{12}=\beta_{12}m_{1},$$

$$\gamma_{21}=\beta_{21}m_{2},$$

$$\gamma_{22}=\alpha_{2}+\beta_{21}m_{1}+2\beta_{22}m_{2},$$

with

$$m_{i}=\int_{\partial R_{+}^{2,\circ }}X_{i}\pi^{*}\left(dX_{1}dX_{2}\right)=\underset{t\to \infty }{\text{lim}}\frac{1}{t}\int_{0}^{t}X_{i}\left(s\right)ds\left(i=1,2\right).$$

Let $A=\left[\begin{array}{ll} \gamma_{11} & \gamma_{12}\\ \gamma_{21} & \gamma_{22} \end{array}\right],\Theta ={\left(\Phi_{1},\Phi_{2}\right)}^{T},B_{1}=\left[\begin{array}{cc} \tau_{11} & 0\\ 0 & \tau_{21} \end{array}\right],B_{2}=\left[\begin{array}{cc} \tau_{12} & 0\\ 0 & \tau_{22} \end{array}\right],\sigma_{ij}=\tau_{i1}\tau_{j1}+\tau_{i2}\tau_{j2}(i,j=1,2)$, and $Y_{i}={\tilde{X}}_{i}-m_{i}(i=1,2)$. Then the system (3.5) becomes

(3.6) $$dY=(AY+\Theta )dt+\left(B_{1}Y+B_{1}m\right)dW_{1}+\left(B_{2}Y+B_{2}m\right)dW_{2}$$

where $Y={\left(Y_{1},Y_{2}\right)}^{T}$ and $m={\left(m_{1},m_{2}\right)}^{T}$. Next, by computation, we have

$$I_{2}⊗A=\left[\begin{array}{cccc} \gamma_{11} & \gamma_{12} & 0 & 0\\ \gamma_{21} & \gamma_{22} & 0 & 0\\ 0 & 0 & \gamma_{11} & \gamma_{12}\\ 0 & 0 & \gamma_{21} & \gamma_{22} \end{array}\right],A⊗I_{2}=\left[\begin{array}{cccc} \gamma_{11} & 0 & \gamma_{12} & 0\\ 0 & \gamma_{11} & 0 & \gamma_{12}\\ \gamma_{21} & 0 & \gamma_{22} & 0\\ 0 & \gamma_{21} & 0 & \gamma_{22} \end{array}\right],$$

$$B_{1}⊗B_{1}=\left[\begin{array}{cccc} \tau_{11}^{2} & 0 & 0 & 0\\ 0 & \tau_{11}\tau_{21} & 0 & 0\\ 0 & 0 & \tau_{21}\tau_{11} & 0\\ 0 & 0 & 0 & \tau_{21}^{2} \end{array}\right],B_{2}⊗B_{2}=\left[\begin{array}{cccc} \tau_{12}^{2} & 0 & 0 & 0\\ 0 & \tau_{12}\tau_{22} & 0 & 0\\ 0 & 0 & \tau_{22}\tau_{12} & 0\\ 0 & 0 & 0 & \tau_{22}^{2} \end{array}\right].$$

Thus $S=I_{2}⊗A+A⊗I_{2}+B_{1}⊗B_{1}+B_{2}⊗B_{2}$, which is equal to

$$S=\left[\begin{array}{cccc} 2\gamma_{11}+\sigma_{11} & \gamma_{12} & \gamma_{12} & 0\\ \gamma_{21} & \gamma_{11}+\gamma_{22}+\sigma_{12} & 0 & \gamma_{12}\\ \gamma_{21} & 0 & \gamma_{11}+\gamma_{22}+\sigma_{21} & \gamma_{12}\\ 0 & \gamma_{21} & \gamma_{21} & 2\gamma_{22}+\sigma_{22} \end{array}\right],$$

and $T=\text{vec}\left(\Theta {\text{M}}^{\text{T}}+{\text{M}\Theta }^{\text{T}}\right)+\left[{\text{B}}_{1}⊗{\;\text{B}}_{1}+{\text{B}}_{2}⊗{\;\text{B}}_{2}\right]\text{vec}\left({\text{M}m}^{\text{T}}+{m\text{M}}^{\text{T}}+{mm}^{\text{T}}\right)$, which is equal to

$$T=\left[\begin{array}{c} 2m_{1}\theta_{1}+\sigma_{11}\left(2M_{1}m_{1}+m_{1}^{2}\right)\\ m_{1}\theta_{2}+m_{2}\theta_{1}+\sigma_{12}\left(M_{2}m_{1}+m_{2}M_{1}+m_{2}m_{1}\right)\\ m_{1}\theta_{2}+m_{2}\theta_{1}+\sigma_{21}\left(M_{1}m_{2}+m_{1}M_{2}+m_{1}m_{2}\right)\\ 2m_{2}\theta_{2}+\sigma_{22}\left(2M_{2}m_{2}+m_{2}^{2}\right) \end{array}\right],$$

in which $M={\left(M_{1},M_{2}\right)}^{T}={\left(EY_{1},EY_{2}\right)}^{T}$ is the solution to the ODE system

(3.7) $$\dot{M}=AM+\Theta ,$$

with the initial value M(0)=u. If we let $Z={\left(EY_{1}^{2},EY_{2}Y_{1},EY_{1}Y_{2},EY_{2}^{2}\right)}^{T}$, then we can smooth out the solutions of (3.6) by looking at the solutions of the following ODE system

(3.8) $$\frac{dZ}{dt}=SZ+T,$$

By using two ODE systems (3.7) and (3.8), we will develop graphical representations of the four resilience measures, which are the instantaneous return rate, the average return rate, the asymptotic resilience, and the convergence rate, for the stochastic system (3.1) of the competitive type over the relevant parameter regimes in the next subsection.

### 3.2. Recovery after pulse perturbation

As a simple illustration of how environmental noise and microbial interactions combine to drive the rate of return of a microbial community to the equilibrium state after a pulse perturbation, we look at a SgLV model of two competitive species (3.1). Here, we assume that two species are in direct competition with each other, so that their interaction strengths $\beta_{12}$ and $\beta_{21}$ are negative.

**Figure 3.**
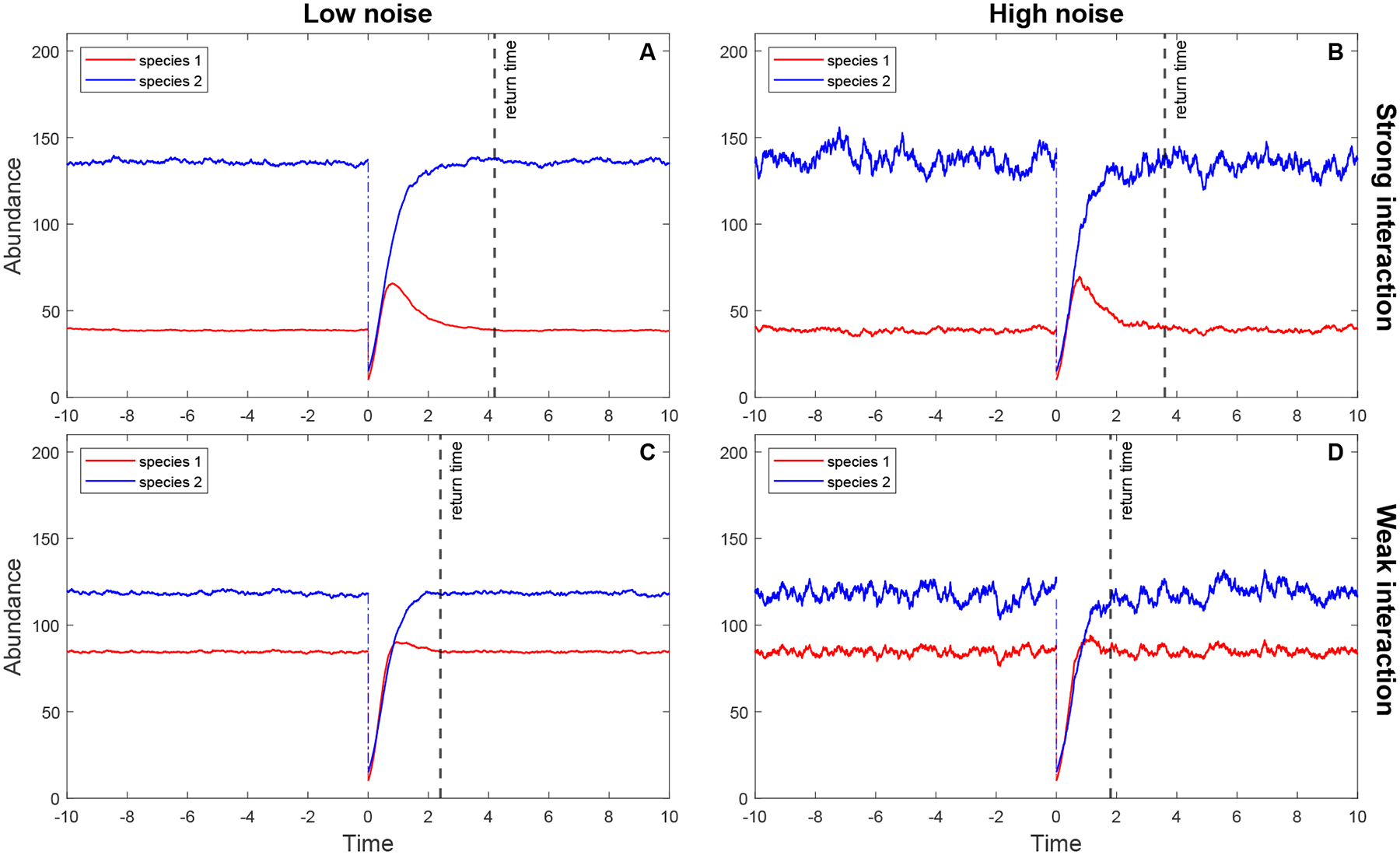
Dynamics of the stochastic system given by (3.1) with four parameter sets. Panel A corresponds to the parameter Set 1 (low noise, strong interaction): $\left(\alpha_{1},\alpha_{2}\right)=(6,4),\left(\beta_{11},\beta_{12},\beta_{21},\beta_{22}\right)=(0.05,-0.03,-0.01,0.027)$, and $\left(\tau_{11},\tau_{12},\tau_{21},\tau_{22}\right)=(0.01,0.015,0.01,0.02)$. Panel B corresponds to the parameter Set 2 (high noise, strong interaction): $\left(\alpha_{1},\alpha_{2}\right)=(6,4),\left(\beta_{11},\beta_{12},\beta_{21},\beta_{22}\right)=(0.05,-0.03,-0.01,0.027)$, and $\left(\tau_{11},\tau_{12},\tau_{21},\tau_{22}\right)=(0.05,0.075,0.05,0.1)$. Panel C corresponds to the parameter Set 3 (low noise, weak interaction): $\left(\alpha_{1},\alpha_{2}\right)=(6,4),\left(\beta_{11},\beta_{12},\beta_{21},\beta_{22}\right)=(0.05,-0.015,-0.01,0.027)$, and $\left(\tau_{11},\tau_{12},\tau_{21},\tau_{22}\right)=(0.01,0.015,0.01,0.02)$. Panel D corresponds to the parameter Set 4 (high noise, weak interaction): $\left(\alpha_{1},\alpha_{2}\right)=(6,4),\left(\beta_{11},\beta_{12},\beta_{21},\beta_{22}\right)=(0.05,-0.03,-0.01,0.027)$, and $\left(\tau_{11},\tau_{12},\tau_{21},\tau_{22}\right)=(0.05,0.075,0.05,0.1)$.

In each panel of Figure 3, two trajectories of the system (3.1) are produced, in which the trajectories before time zero represent the equilibrium states of the two species and the trajectories after time zero are the recovery dynamics of the two species following a pulse perturbation at time zero. The difference between Figure 3A and Figure 3C is due to the difference in the interaction strength $\beta_{12}$, which measures the strength of interspecific competition of Species 2 on Species 1. In Figure 3A, $\beta_{12}$ is twice as large as in Figure 3C, while the noise intensities are the same in both plots. In contrast, the difference between Figure 3A and Figure 3B is caused by the difference in the noise intensities of two white noises: In Figure 3B, the noise intensities of $\frac{dW_{1}}{dt}$ and $\frac{dW_{2}}{dt}$ are five times those in Figure 3A, while the interaction strengths between species are the same, leading to greater fluctuations in the species’ abundances. Figure 3D differs from Figure 3A in that the noise intensities increase by a factor of 5 and the interaction strength $\beta_{12}$ decreases by a factor of 2.

Resilience in a pulse-perturbed stochastic system can be illustrated by this example. When comparing Figure 3A and Figure 3C, we observe that decreasing the interaction strength $\beta_{12}$ reduces the return time of the system to the equilibrium state after a pulse perturbation. That is, lower competition between two species intensifies the resilience of the system under the same environmental regime. When comparing Figure 3A and Figure 3B, increasing the noise intensities decreases the time required for recovery trajectories to return to an equilibrium state. In this example, it appears that increasing environmental noise actually increases stability, which contrasts with the intuition that increasing noise destabilizes the stability of the system [21–23]. Figure 3D shows the combined effect of environmental noise and interactions between species on the resilience of a stochastic system. In the next subsection, we will use our theoretical framework to test our observations in Figure 3.

### 3.3. Noise drives resilience

**Figure 4.**
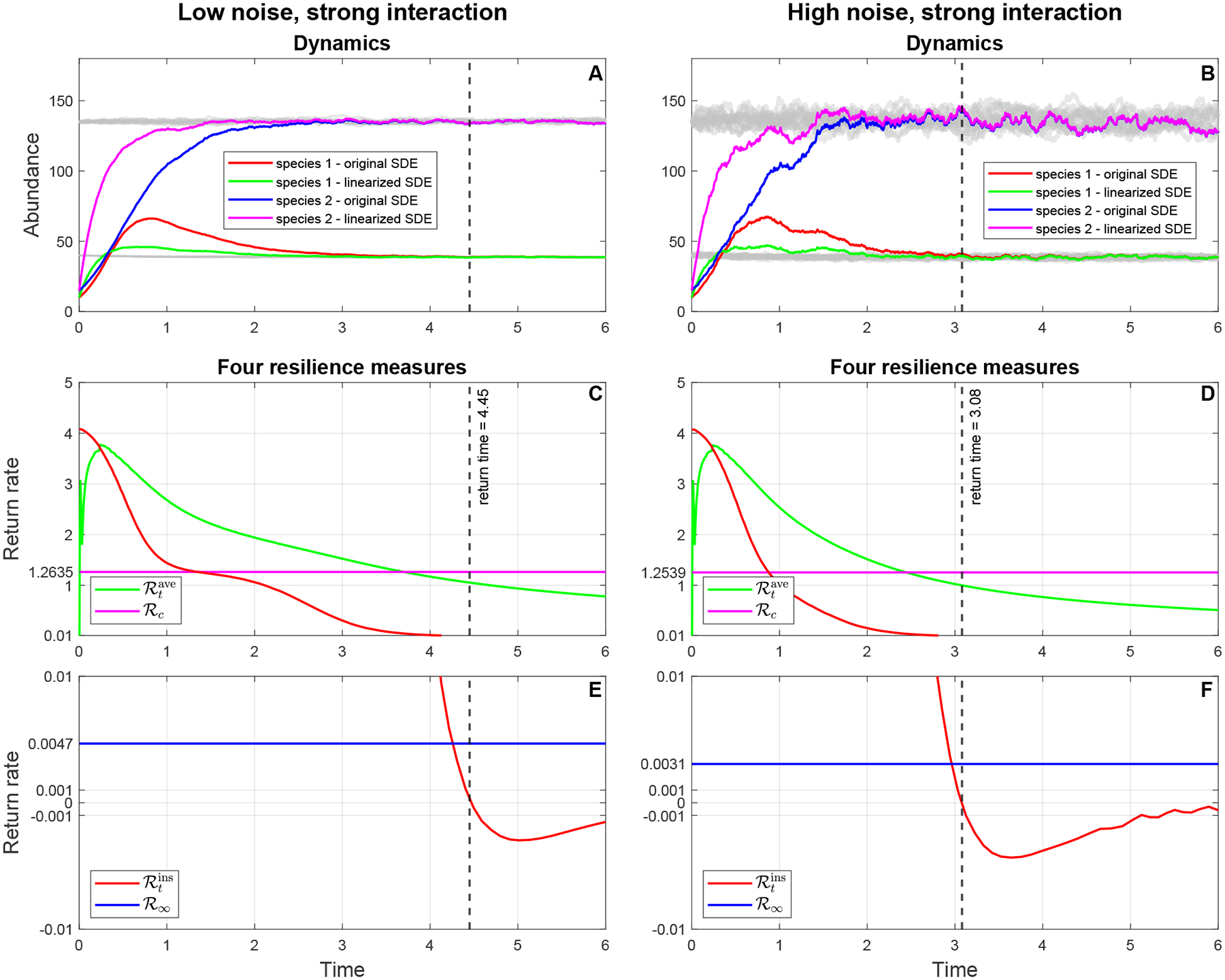
Recovery dynamics (Panels A and B) of both the linearized system (3.1) and the original system (3.5) following the same pulse perturbation (10, 15), and the dynamics of four resilience measures (average return rate, rate of convergence (Panels C and D) and instantaneous return rate, asymptotic resilience (Panels E and F)) in two scenarios of parameter sets 1 and 2.

**Figure 5.**
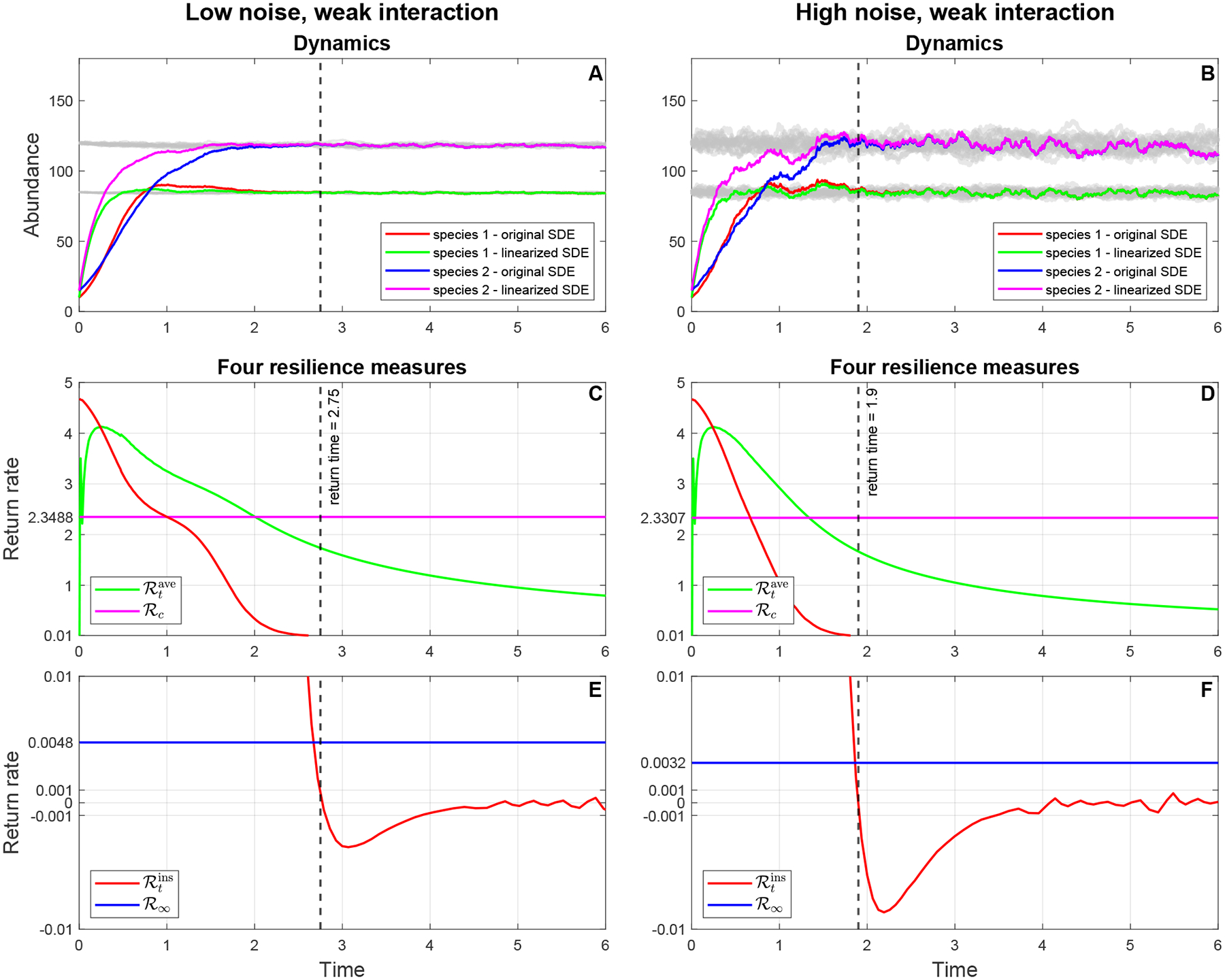
Recovery dynamics (Panels A and B) of both the linearized system (3.1) and the original system (3.5) following the same pulse perturbation (10,15), and the dynamics of four resilience measures (average return rate, rate of convergence (Panels C and D) and instantaneous return rate, asymptotic resilience (Panels E and F)) in two scenarios of parameter sets 3 and 4.

To demonstrate the usefulness of our theoretical framework developed above, we apply the procedure to calculate the resilience of a SgLV (described in Figure 2) to the simulated data in Figure 3. If we observe Figure 3 in Section 2, the recovery trajectories of two species return to an equilibrium state at time 4.2 after a pulse perturbation at time zero in Panel A. Reducing the interaction strength $\beta_{12}$ by 2-fold relative to Panel A, Panel C shows that the return time to the equilibrium state is reduced to approximately 2.4 after the same pulse perturbation as in Panel A at time zero. This means that the microbial community in Panel C is more resilient than that in Panel A. Increasing the noise intensities five times compared with Panel A, the return time to the equilibrium state in Panel B decreases to some time around 3.6 with the same perturbation as in Panel A. In other words, the microbial community in Panel C is more resilient than that in Panel A. This shows that environmental noise influences the resilience of the microbial community. Panel D shows the fastest return time, which is approximately 1.8, to the equilibrium state among communities when we decrease the interaction strength $\beta_{12}$ two times and increase the noise intensities five times. Below, we calculate four resilience measures for each panel of Figure 3 to confirm our observation that the resilience of the microbial community is driven by environmental noise and to disentangle the sources of the effects of environmental fluctuations on the resilience of a microbiome system. To calculate the resilience measures, the first step is to linearize the stochastic system (3.1) at its stationary distribution $\pi^{*}$, and then we can obtain the linearized stochastic system (3.5), where all coefficients $\Phi_{i},\gamma_{ij}$, and $m_{i}(i,j=1,2)$ are computed and given in Table 1. Note that ${\left(m_{1},m_{2}\right)}^{T}$ is the expectation of the stationary distribution $\pi^{*}$. Figures 4A, 4B, 5A, and 5B show that the stochastic dynamics of both the original stochastic system (3.1) and the linearized stochastic system (3.5) converge to the equilibrium state characterized by the stationary distribution $\pi^{*}$ following the same pulse perturbation at time 0 in four scenarios corresponding to the four parameter sets in Table 1. In Figure 4A, we see that the trajectories of the linearized system approach and merge into those of the original system between time 4 and 4.5, while the time in Figure 4B is between 3 and 3.5. In Figure 5A, the merging time of the trajectories of both systems is between 2.5 and 3. The fastest merging time between 1.5 and 2 is shown in Figure 5B. Thus, the patterns of the return time to the equilibrium state in both the original system and the linearized system when applied to the same pulse perturbation are the same, even though the dynamics of the trajectories of both systems are quite different before the merging time. Therefore, we are able to utilize a much simpler linearized system (3.5) to investigate the resilience of a microbial community whose temporal dynamics are captured by the original system (3.1).

Next, we transform the linearized system (3.5) into the two linear ODE systems (3.7) and (3.8) by, respectively, finding the first and second moment of solutions and then using vectorization in linear algebra. Then four resilience measures ${ℛ}_{t}^{\text{ins}},{ℛ}_{t}^{\text{ave}},{ℛ}_{c}$, and ${ℛ}_{\infty }$ are calculated on the basis of W(t), the mean square difference between the solution of the linearized system (3.5), the expectation of the stationary distribution $\pi^{*}$, and the mean square stability matrix S. Note that the mean square stability matrix S and W(t) can be calculated from the linearized coefficients shown in Table 1. The last four panels of Figure 4 and Figure 5 show the dynamics of four resilience measures over time in the four scenarios above.

Two resilience measures ${ℛ}_{t}^{\text{ins}}$ and ${ℛ}_{t}^{\text{ave}}$, which are deterministic functions of time, can help us track the short-term behavior of lnW(t). When ${ℛ}_{t}^{\text{ins}}>0,\frac{d}{dt}\;\text{ln}\;W(t{)}^{1/2}<0$, which implies that lnW(t) is decreasing to its equilibrium point at time t. In other words, when ${ℛ}_{t}^{\text{ins}}>0$, the solution $\tilde{X}(t)$ of the linearized system (3.5) moves closer to the equilibrium state $\pi^{*}$ at time t. By the same argument, when ${ℛ}_{t}^{\text{ins}}<0,$ lnW(t) is increasing, and hence the solution $\tilde{X}(t)$ moves away from the equilibrium state $\pi^{*}$ at time t. In terms of ${ℛ}_{t}^{\text{ave}}$, if ${ℛ}_{t}^{\text{ave}}>0$, then lnW(t)<lnW(0), which means that the solution $\tilde{X}(t)$ comes closer to the equilibrium state at time t, since the end of the perturbation at time 0. If ${ℛ}_{t}^{\text{ave}}<0$, then the solution $\tilde{X}(t)$ moves far away from the equilibrium state since the end of the perturbation. The deterministic function ${ℛ}_{t}^{\text{ins}}$ measures the rate of change in lnW(t). So we can use it to track when lnW(t) comes back to its equilibrium point after the perturbation is applied. It is straightforward to see that the time when lnW(t) reaches its equilibrium point (which is a constant) is the time when ${ℛ}_{t}^{\text{ins}}$ crosses 0. Therefore, we can estimate the return time of the solution $\tilde{X}(t)$ to the equilibrium state $\pi^{*}$ when the first time ${ℛ}_{t}^{\text{ins}}$ crosses 0. In the last four panels of Figure 4, the return time to the equilibrium state decreases from 4.45 to 3.08 when the noise intensities are increased by five times. The same decreasing pattern in the return time occurs in the last four panels of Figure 5, which is that the return times go down from 2.75 to 1.9. This pattern can be explained by the physical meaning of W(t). By our definition in Section 2.4, W(t) is the sum of all the variances of the solution components of the linearized system (3.5), which measures how far the solution of the linearized system (3.5) is from the equilibrium state $\pi^{*}$. Then high noise makes the equilibrium state $\pi^{*}$ have higher variance so that there is less recovery required following perturbation to return to $\pi^{*}$. So, the return to the equilibrium state $\pi^{*}$ is actually faster than in the system with high noise than with low noise.

Two resilience measures ${ℛ}_{c}$ and ${ℛ}_{\infty }$, which are constants, can be used to predict the resilience of the stochastic system (3.1) by looking at the gap ${ℛ}_{c}-{ℛ}_{\infty }$ and how fast the average return rate ${ℛ}_{t}^{\text{ave}}$ enters this gap. In Table 2, when we increase the noise five times, the gap ${ℛ}_{c}-{ℛ}_{\infty }$ decreases from 1.2588 (parameter Set 1: low noise, strong interaction) to 1.2508 (parameter Set 2: high noise, strong interaction). From Panels C and D in Figure 4, we also observe that the time when the average return rate ${ℛ}_{t}^{\text{ave}}$ moves into the gap between ${ℛ}_{c}$ and ${ℛ}_{\infty }$ is shorter when the noise increases. This pattern is the same as we move from parameter Set 3 (low noise, weak interaction) to parameter Set 4 (high noise, weak interaction). The pattern of a decreased gap implies shorter for moving into the time gap, caused by increasing noise, which can be elucidated by looking at the mathematical meanings of the two resilience measures ${ℛ}_{c}$ and ${ℛ}_{\infty }$. First, the quantity ${ℛ}_{c}$ measures how fast the covariant matrix P(t), which is the solution of the system (2.19), converges to its equilibrium state. Basically, ${ℛ}_{c}$ equals the negative half of the real part of the largest eigenvalue of the mean square stability matrix S, which means that ${ℛ}_{c}$ tells us about the smallest convergence rate among the convergence rates of the elements of the covariant matrix P(t). While ${ℛ}_{\infty }$ can be considered as an equilibrium state of the average return rate ${ℛ}_{t}^{\text{ave}}$, and so ${ℛ}_{\infty }$ approximately equals the smallest convergence rate among the convergence rates of the diagonal elements of the covariance matrix P(t). Therefore, the gap ${ℛ}_{c}-{ℛ}_{\infty }$ is always positive when noise appears in the original system (3.1). Without noise, this gap will be zero, and hence these two resilience measures coincide. Our simulations (Figures 4 and 5) and calculations (Table 2) demonstrate that increasing noise will decrease the gap ${ℛ}_{c}-{ℛ}_{\infty }$, which, in turn, decreases the time when ${ℛ}_{t}^{\text{ave}}$ enters the gap. This implies that increasing the level of noise increases the resilience of the system.

## 4. Discussion

The concept of resilience has been used for a long time in community ecology, although it is much less developed for microbial systems, even though resilience is at the center of many pressing questions in microbiome research. Microbiome communities differ from macroecological communities in at least two important ways that impact the modeling of ecological stability. First, while stability metrics in macroecological systems have been developed for first-order multivariate autoregressive models [21, 22], the nonlinear generalized Lotka–Volterra model is more commonly used in microbiome studies. This kind of model has been applied to study time-series inference and stability in intestinal microbiota [7, 38], to model primary succession of murine gut communities [35], and to capture dynamics of complex microbial metapopulations in aquatic environments [36, 37]. Second, and perhaps more importantly, microbial time-series data are incredibly noisy. Some of this noise is measurement error due to the sampling and sequencing processes involved in data collection. These challenges have been documented in various microbial systems, from cheese communities [40] to human microbiome studies with dense temporal sampling [41,42]. Furthermore, noise arises in experimental studies of gut microbiota reconstitution and synthetic community design [43,44], as well as in investigations of microbiome dynamics at different timescales [45,46]. But there is also intrinsic noise in the system, with sources including fluctuations in temperature, different local concentrations of species, or slight variations among individuals.

Currently, existing definitions of resilience are based on the theory of ordinary differential equations or difference equations, so all existing stability metrics to measure resilience are not functions of noise. In this paper, we have built a general framework to allow stochasticity to be deeply involved in the dynamics of microbial communities. Specifically, we have proposed an SgLV model to describe the temporal dynamics of a microbial community with white noise added to each interaction. We then developed a general theoretical framework for how to calculate four resilience measures based on the SgLV model. Each measure captures a different facet of stability and, together, they give a picture of resilience.

All four resilience measures are inherently functions of noise, since they are defined in terms of the ODE system established by the variance matrix of the linearized SDE system. Therefore, all four measures can be manipulated by the magnitude of the noise. One surprising result to emerge from our framework is that the return to the original stationary state is actually faster in the system with high noise than with low noise. This is primarily driven by the fact that the stationary distribution has higher variance in the system with high noise, so that there is less recovery required following a perturbation to return to the stationary distribution.

The next step of this work is the possibility of applying this general framework to time-series data from real-world microbiomes. This application involves a fundamental question regarding the identifiability and estimability of the parameters within our proposed stochastic generalized Lotka–Volterra (SgLV) model. Parameter identifiability remains a significant challenge, particularly for complex hierarchical models involving microbiome dynamics. While traditional approaches to identifiability, such as those described in Browning 2020 [47] and in Remien 2021 [48], focus primarily on deterministic systems, fewer methodologies explicitly address stochastic models. To address this critical gap, methodologies such as data cloning, as described by Lele et al., 2010 [49] may be particularly valuable. Although originally designed for generalized linear mixed models, data cloning could be adapted to our SgLV framework to provide a practical graphical test for assessing parameter estimability. This approach involves generating multiple replicated datasets (clones) from microbiome time-series data and using Markov Chain Monte Carlo (MCMC) methods to sample from the resulting posterior distributions. By assessing whether the parameters have degenerate posterior distributions as the number of clones increases, we can determine whether each parameter—or specific functions of parameters, such as the resilience metrics proposed in our study—is estimable. Such a rigorous assessment of estimability would significantly enhance the reliability of the scientific conclusions drawn from applying our framework to real-world microbiome data.

An important direction of future research is the application of our proposed stochastic generalized Lotka–Volterra (SgLV) framework and resilience measures to practical scenarios in microbiome studies. For example, predicting the recovery dynamics of microbial communities after perturbations such as single-dose antibiotic treatments represents a significant area of potential application. Recent studies, including those by Hayashi et al., 2024 [24] and Ponciano et al., 2024 [25], have demonstrated that microbial community compositions can shift between alternative stable states under environmental perturbations, influenced by both deterministic selection and stochastic drift. Using our developed resilience metrics, researchers could quantify the microbiome’s rate and likelihood of returning to a “healthy”or pre-perturbation state, providing a valuable predictive tool for microbiome resilience. This approach could significantly aid experimental design, inform intervention strategies, and guide the interpretation of the microbiome’s recovery patterns following ecological disturbances.

Nevertheless, applying our SgLV framework and resilience metrics to real-world microbiome data requires careful consideration of several microbial-data-specific challenges. Microbiome datasets frequently suffer from the undersampling of rare taxa, which can lead to inaccurate abundance estimates and consequently affect the reliability of the calculated resilience measures. Additionally, relative abundance data, as commonly obtained through sequencing technologies, complicate the accurate estimation of interaction parameters because these data lack information on the absolute abundances. As emphasized by Ponciano et al., 2024 [25], addressing these challenges might involve combining sequencing-based relative abundances with absolute abundance quantification methods, such as quantitative polymerase chain reaction (PCR), to improve the estimation accuracy of interaction coefficients. Additionally, sparse temporal sampling can obscure rapid microbial dynamics, potentially reducing the reliability of resilience predictions. Future studies might employ robust parameter estimation methods such as data cloning (Lele et al., 2010 [49]) to assess parameter estimability rigorously and thus enhance confidence in resilience measures calculated from real-world microbiome time-series data.

Recently, Flamant et al., 2020 [50] introduced an innovative approach using neural network solution bundles to approximate the solutions of ordinary differential equations, which could be extended effectively to stochastic generalized Lotka–Volterra (SgLV) models for microbiome studies. In this approach, neural networks are trained to approximate solution bundles of differential equations over a broad parameter space, enabling efficient exploration of model dynamics and rapid fitting to empirical data. Specifically, once trained, these neural network solution bundles could quickly approximate the trajectories predicted by the SgLV model, significantly reducing the computational overhead compared with traditional numerical solvers. When applied to real-world microbiome time-series data, this methodology could effectively address common data challenges. For instance, by efficiently handling sparse temporal sampling, neural network approximations could infer microbiome dynamics even when the data resolution is limited. Furthermore, the flexibility of neural networks might better accommodate biases arising from relative abundance data, potentially integrating with methods used to infer absolute abundances. Lastly, the approach could also help mitigate the challenges arising from undersampling rare taxa by efficiently exploring parameter spaces to identify robust solutions despite incomplete information on abundance. Thus, adopting neural network solution bundles presents a promising strategy for reliably applying the SgLV framework to complex microbiome datasets.

## Figures and Tables

**Table 1.** Coefficients of the linearized stochastic system (3.5) are computed in four scenarios that correspond to the four parameter sets in Figure 3.

	Parameter set 1	Parameter set 2	Parameter set 3	Parameter set 4
$m_{1}$	38.7470	38.8413	84.4804	84.4508
$m_{2}$	135.3518	134.9853	118.2069	117.8869
$\Phi_{1}$	0.0813	0.3254	0.2431	0.7731
$\Phi_{2}$	0.4263	1.6171	0.3562	1.3958
$\gamma_{11}$	−1.9353	−1.9337	−4.2211	−4.2134
$\gamma_{12}$	−1.1624	−1.1652	−1.2672	−1.2668
$\gamma_{21}$	−1.3535	−1.3499	−1.1821	−1.1789
$\gamma_{22}$	−3.6062	−3.5876	−3.1492	−3.1318

**Table 2.** The difference ${ℛ}_{c}-{ℛ}_{\infty }$ computed from the linearized coefficients in Table 1 in four scenarios corresponding to the four parameter sets in Figure 3.

Low noise, strong interaction	High noise, strong interaction
${ℛ}_{c}^{1}=1.2635$	${ℛ}_{c}^{2}=1.2539$
${ℛ}_{\infty }^{1}=0.0047$	${ℛ}_{\infty }^{2}=0.0031$
${ℛ}_{c}^{1}-{ℛ}_{\infty }^{1}=1.2588$	${ℛ}_{c}^{2}-{ℛ}_{\infty }^{2}=1.2508$
Low noise, weak interaction	High noise, weak interaction
${ℛ}_{c}^{3}=2.3488$	${ℛ}_{c}^{4}=2.3307$
${ℛ}_{\infty }^{3}=0.0048$	${ℛ}_{\infty }^{4}=0.0032$
${ℛ}_{c}^{3}-{ℛ}_{\infty }^{3}=2.3440$	${ℛ}_{c}^{4}-{ℛ}_{\infty }^{4}=2.3275$
